# Dynamic change of polarity in spread through air spaces of pulmonary malignancies

**DOI:** 10.1002/path.6382

**Published:** 2025-01-13

**Authors:** Yoshiaki Matsuura, Kunishige Onuma, Roberto Coppo, Hiroyuki Uematsu, Jumpei Kondo, Aya Miyagawa‐Hayashino, Naoko Takeda‐Miyata, Kenji Kameyama, Tatsuo Furuya, Satoru Okada, Masanori Shimomura, Masayoshi Inoue, Masahiro Inoue

**Affiliations:** ^1^ Department of Clinical Bio‐resource Research and Development, Graduate School of Medicine Kyoto University Kyoto Japan; ^2^ Divison of Thoracic Surgery, Department of Surgery, Graduate School of Medical Science Kyoto Prefectural University of Medicine Kyoto Japan; ^3^ KBBM Inc. Kyoto Japan; ^4^ Department of Molecular Biology and Clinical Investigation, Graduate School of Medicine Osaka University Osaka Japan; ^5^ Department of Surgical Pathology Kyoto Prefectural University of Medicine Kyoto Japan

**Keywords:** spread through air spaces, STAS, lung cancer, colorectal cancer, organoid, metastasis, polarity, follistatin‐like protein 1, FSTL1, TGF‐β1

## Abstract

Spread through air spaces (STAS) is a histological finding of lung tumours where tumour cells exist within the air space of the lung parenchyma beyond the margin of the main tumour. Although STAS is an important prognostic factor, the pathobiology of STAS remains unclear. Here, we investigated the mechanism of STAS by analysing the relationship between STAS and polarity switching *in vivo* and *in vitro*. Histopathological analysis revealed that apical membranes were observed outside the STAS lesions around colorectal cancer (CRC) lung metastases and lung adenocarcinomas. When apical‐out CRC organoids were administered intratracheally to mice, the organoids had greater metastatic potential than did single cells. To investigate the pathobiology of STAS, we established an *in vitro* model of STAS in which CRC or lung cancer organoids were co‐cultured with 2D‐cultured mouse airway epithelial organoids (2D‐MAOs). Adhesion of cancer organoids to 2D‐MAOs was much less than to type I collagen or endothelial cells, suggesting a protective role of the airway epithelium against adhesion. Loss of the apical membrane of CRC organoids at the contact surface with 2D‐MAOs after adhesion was responsible for establishing adhesion. When airway epithelium was stimulated by transforming growth factor beta 1 (TGF‐β1), adhesion of CRC organoids was enhanced. Among TGF‐β1‐induced genes in airway epithelium, follistatin‐like protein 1 (FSTL1) increased CRC organoid adhesion by promoting loss of the apical membrane. These results suggested that TGF‐β1‐induced FSTL1 may promote metastatic progression of STAS by altering the polarity status. Elucidating the mechanism of STAS could contribute to the improvement of survival in patients with pulmonary malignancies associated with STAS. © 2025 The Author(s). *The Journal of Pathology* published by John Wiley & Sons Ltd on behalf of The Pathological Society of Great Britain and Ireland.

## Introduction

Spread through air spaces (STAS) was introduced as one of the prognostically important histological findings of lung tumours in the 2015 WHO classification [1, 2]. The WHO classification defines STAS as ‘tumour cells within the air space of the lung parenchyma beyond the margin of the main tumour’ [2]. The aetiology of this disease is not fully understood, but the high rate of local recurrence suggests the possibility of metastasis via the airways [3, 4]. STAS is a factor predictive of prognosis even in completely resected early‐stage lung adenocarcinoma [5]. It has also been reported that locoregional recurrence including ipsilateral pulmonary metastases is more frequently observed in STAS‐positive lung cancer patients after sub‐lobar resection including segmentectomy and wedge resection [6, 7].

The lung is the second most common distant metastatic site of colorectal cancer (CRC) following the liver, and its control is a crucial factor in the treatment of CRC. Surgical resection is the first choice for pulmonary metastatic lesions in CRC, but a relatively high rate of local recurrence has been reported [8]. Since STAS is associated with a poor prognosis also in pulmonary metastases of CRC [9, 10], STAS may be involved in local recurrence.

It has been reported that circulating tumour cell (CTC) clusters have a greater advantage than do single cells in cancer metastasis, since CTC clusters have superior cell survival ability, cancer stemness retention, and immune evasion [11, 12]. CTC clusters metastasise more efficiently *in vivo* and are associated with a worse prognosis and lower overall survival of patients with cancer than are single CTCs. Thus, CTC clusters released into the airway from the main tumour in the lung may be a source of STAS.

Organoids generated from differentiated CRCs change the direction of apico‐basal polarity depending on the culture conditions. When cultured in suspension, an apical membrane forms on the outside of the organoid (apical‐out). However, when cultured in Matrigel or type I collagen, the apical membrane covers the lumen that forms inside the organoids (apical‐in) [13]. Apical‐out status is observed clinically in the areas of micro‐vessel invasion [13] and micropapillary carcinoma lesions of CRC [14], as well as in cancer cell clusters in ascites of ovarian cancer [15]. Polarity switching occurs quickly in both directions (between apical‐in and apical‐out), and plays an important role in experimental liver metastasis of CRC [13] and peritoneal dissemination of ovarian cancer [15]. The relevance and role of polarity switching in STAS have not been elucidated.

Owing to advances in organoid technology, airway organoids can now be cultured for long periods of time and differentiated into a wide variety of cells [16]. Airway organoids have been applied as a model for various respiratory diseases including genetic and infectious diseases [17]. In this study, an *in vitro* model of STAS was established using co‐cultures of cancer organoids and airway epithelium, and the process of adhesion of cancer cell clusters to the airway epithelium and the clearance of airway epithelium by cancer cell clusters were examined. Elucidating the mechanism of STAS will contribute to the development of therapies to prevent STAS and its associated metastases.

## Materials and methods

Detailed methods for cell preparation and culture, cell viability assays, plasmids, microarray analysis, and western blotting are provided in Supplementary materials and methods.

### Ethics statement

The study was approved by the Institutional Ethics Committees at Kyoto University (R1575, R2444), Kyoto Prefectural University of Medicine (ERB‐C‐1807), and Osaka International Cancer Institute (1803125402). Fresh surgical samples from patients were obtained with the patients’ informed consent. The animal studies were approved by the Institutional Animal Care and Use Committee of Kyoto University (18564).

### *In vivo* experiments

The luciferin‐expressing C45 CRC organoid, C45/Luc2, was passaged by partial dissociation of the organoids. After incubation for at least 3 days, organoids between 40‐ and 70‐μm filters were collected. The CRC organoids were counted and *n* = 1,000 organoids were suspended in 20 μl of PBS and then administered intratracheally to male, 15‐week‐old, NOD/SCID mice (CLEA Japan, Tokyo, Japan). Organoids were dissociated into single cells by pipetting *n* = 100 times after 10 min of 0.25% trypsin/EDTA treatment. The percentage of dead cells after dissociation into single cells was assessed using Trypan Blue staining and was about 10%. 3.3 × 10^4^ living single cells, equivalent to 1,000 organoids, were administered into the trachea of mice. For *in vivo* bioluminescence imaging, 2 mg of d‐luciferin (VivoGlo Luciferin; Promega, Madison, WI, USA) was dissolved in 100 μl of PBS and injected intraperitoneally (i.p.) into anaesthetised mice 5 min before imaging. Bioluminescence was measured using an IVIS system (Xenogen Corporation, Alameda, CA, USA).

### Histological analyses

Haematoxylin and eosin (H&E) staining and immunofluorescence staining were performed using formalin‐fixed, paraffin‐embedded tissue sections of clinical and xenograft tumours as described previously [15]. The primary antibodies used are listed in supplementary material, Table S1. Images were acquired with a BX50 microscope using the CellSens standard imaging software (Olympus, Tokyo, Japan). For *in vitro* assays, dissociated mouse airway epithelial organoids (MAOs) were plated in glass‐bottom dishes (Matsunami, Osaka, Japan) coated with Cellmatrix Type IA (Nitta Gelatin Inc., Osaka, Japan) at 1.5 × 10^5^ cells/cm^2^. Immunostaining for confluent 2D‐MAOs was performed on day 7 after fixation with 4% paraformaldehyde. Images were acquired using a Leica TCS SPE confocal microscope (Leica Microsystems, Wetzlar, Germany). Details of the confocal imaging for assessing interactions between CRC organoids and mouse airway epithelial organoids (MAOs) are provided in Supplementary materials and methods.

### Quantitative analysis of polarity status

Paraffin‐embedded patient tumours were sectioned and immunostained for villin (for CRC) and ezrin for lung adenocarcinoma. STAS polarity status was classified into four categories: Complete (apical proteins present in more than 50% of the outermost membrane of the STAS region but not inside the STAS region), Partial (apical proteins present in less than 50% of the outermost membrane of the STAS region but not inside the STAS region), Mixed (apical proteins present on both the outermost membrane of the STAS region and the luminal surface inside the STAS region), and None (no apical proteins detected). For *in vitro* analysis, C45 organoids were cultured in suspension (floating) or in 5% Matrigel (in‐gel) for 48 h. Paraffin‐embedded organoids were sectioned and immunostained for villin. The polarity status of the organoids was classified into four groups: apical‐out (villin located only at the surface of an organoid), apical‐in (villin located only at multiple lumina inside an organoid), mixed (villin located both at the surface and at multiple lumina inside an organoid), and none (villin not detected). Multiple organoids were analysed and categorised, and the proportion of each polarity status was calculated.

### Quantitative analysis of local apical membrane loss

The apical membranes of C45 organoids, at the interface with MAOs, were evaluated at specific time points using Z‐stack images of confocal microscopy. MAOs were stained with CellTracker Red and co‐cultured with C45 organoids expressing glycosylphosphatidylinositol‐green fluorescent protein (GPI‐GFP). The interface was classified into two regions: one with green fluorescence at the organoid surface (FOS) and one without green fluorescence at the organoid surface (NFOS). The lengths of the FOS and NFOS regions were measured using ImageJ (NIH, Bethesda, MD, USA). The NFOS ratio was calculated using the following formula: NFOS/FOS + NFOS.

### Assays for evaluating interactions between CRC organoids and MAOs

Adhesion, detachment, and clearance assays are illustrated in supplementary material, Figure S1. 2D‐MAOs were prepared as follows. MAOs were dissociated into single cells and suspended in 100 μl of MAO medium, and 5 × 10^4^ single cells per well were plated on 96‐well plates coated with Cellmatrix Type IA (day 0). Medium was replaced on day 3 and the confluent 2D‐MAOs were subjected to the assays on day 7. For assays with human hepatic sinusoidal endothelial cells (HHSECs), 5 × 10^3^ single cells were plated on fibronectin‐coated 96‐well plates. HHSECs were subjected to the assays on day 3.

For the 2D‐MAO adhesion assay, cancer organoids 40–100 μm in diameter and confluent 2D‐MAOs were co‐cultured in 96‐well plates for 24 h (organoid counts were C45: 100; MCL1 apoptosis regulator: 30; others: 50) in the co‐culture medium (1:1 mixture of MAOs and CRC organoid medium). The culture medium was then removed, and the wells were gently washed twice with 100 μl of PBS warmed to 37 °C. The number of organoids in each well was counted before and after washing using a Cell3iMager duos imager (SCREEN, Kyoto, Japan), and the adhesion rate was calculated as shown in supplementary material, Figure S1. The type I collagen adhesion assay was performed as above without MAOs. The HHSEC adhesion assay was performed as above using HHSECs instead of MAOs in the co‐culture medium (1:1 mixture of HHSECs and CRC organoid medium). For the detachment assay, *n* = 200 organoids and 2D‐MAOs were co‐cultured and washed as in the adhesion assay. Then 100 μl of fresh co‐culture medium was added and incubated for another 24 h. The wells were washed as described above, and the total number of organoids in each well was counted before and after washing. The detachment rate was calculated as shown in supplementary material, Figure S1. For the clearance assay, 2D‐MAOs were stained with CellTracker Green CMFDA Dye (Thermo Fisher Scientific, Waltham, MA, USA) for 1 h and washed twice with 100 μl of PBS. Then *n* = 30 C45/mCherry organoids were plated. Co‐culture and washing were performed as in the adhesion assay. The clearance regions within 2D‐MAOs were detected as regions with no fluorescence signals below the organoids. The clearance rate was calculated as shown in supplementary material, Figure S1.

The assays using cytokines, dasatinib, and recombinant human FSTL1 (rhFSTL1; Peprotech, Cranbury, NJ, USA) are illustrated in supplementary material, Figure S2. For assays with cytokines, 20 ng/ml cytokines was added to the MAO medium on day 3; to the cancer organoid medium on day 5; and to the co‐culture medium on day 7. For assays with TGF‐β1 pre‐treatment, 20 ng/ml TGF‐β1 was added to MAO medium lacking noggin and A83‐01 on day 3, and to the cancer organoid medium on day 5. For assays with dasatinib pre‐treatment, 10 μm dasatinib was added to the cancer organoid medium on day 5. For assays with rhFSTL1, MAOs or cancer organoids were pre‐treated with 500 ng/ml rhFSTL1 on day 6. Co‐culture was started on day 7 in all experiments.

### Statistical analyses

Statistical analyses were conducted using GraphPad Prism version 10 (GraphPad Software Inc., San Diego, CA, USA). Data are expressed as mean ± SD. Significance was tested using an unpaired *t*‐test for single comparisons and one‐way analysis of variance followed by Tukey's test for multiple comparisons. The Mann–Whitney *U*‐test, a non‐parametric test, was used for non‐normally distributed assay results. *p* < 0.05 was considered significant.

## Results

### STAS showed apical‐out polarity status

**Figure 1:**
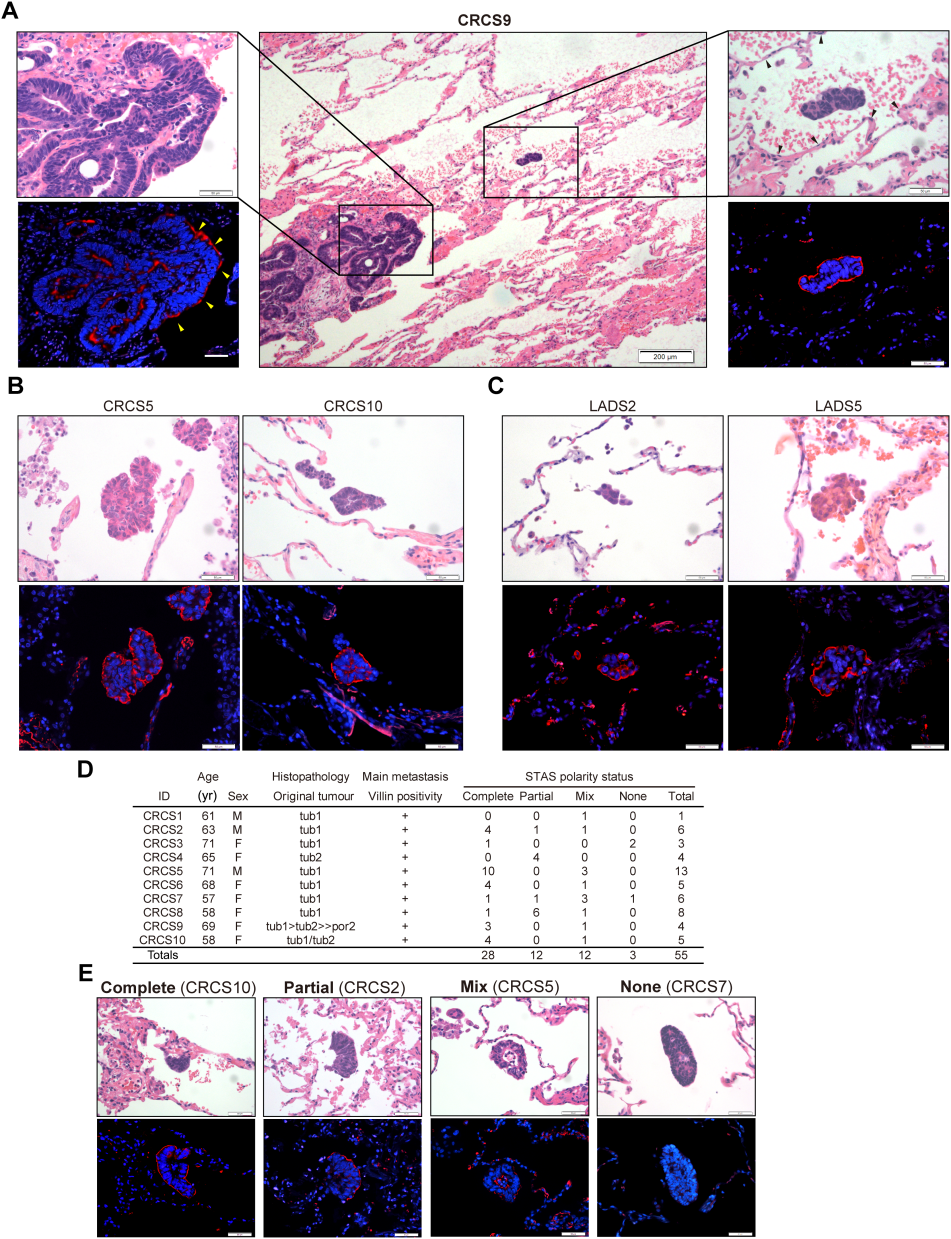
Polarity status of colorectal cancer (CRC) lung metastasis with STAS. (A) Polarity status of a main tumour and STAS of CRC lung metastasis in case CRCS9. Haematoxylin and eosin (H&E) staining (centre; scale bar: 200 μm). Higher magnification of the main tumour (left panels) and STAS region (right panels). Scale bars: 50 and 200 μm for the middle panel. Immunostaining for villin (red) with DAPI counterstain (blue). The alveolar epithelium and the portion of tumour exposed in the air space are indicated by black and yellow arrowheads, respectively. (B) STAS regions of CRC lung metastases in case CRCS5 (left panels) and case CRCS10 (right panels), showing H&E staining (top panels) and immunostaining [villin (red) and DAPI (blue)] (bottom panels). Scale bar: 50 μm. (C) STAS regions of lung adenocarcinoma in case LADS2 (left panels) and case LADS5 (right panels) showing H&E staining (top panels) and ezrin (red) and DAPI (blue) immunostaining (bottom panels). Scale bar: 50 μm. (D) Summary of polarity status of the STAS regions in lung metastases from well‐ or moderately differentiated CRC. (E) Representative images of each polarity category of CRC STAS: Complete (case CRCS10), Mix (case CRCS5), Partial (case CRCS2), and None (case CRCS7). H&E staining (top panels) and immunostaining [villin (red) and DAPI counterstain (blue)] (bottom panels) are shown for each polarity status category. Scale bar: 50 μm.

To clarify the role of polarity in STAS‐induced metastasis, the polarity status of CRC lung metastases with STAS was investigated. The main metastatic tumour showed apical‐in polarity (supplementary material, Figure S3A) similar to the original tumours. In contrast, the exposed portion of the main metastatic tumour in the air space showed apical‐out polarity (Figure 1A and supplementary material, Figure S3B). STAS regions, observed as small clusters of cancer cells in the air space away from the main metastatic tumour, showed apical‐out polarity (Figure 1A). Apical‐out polarity was also observed in other cases of STAS of CRC lung metastasis (Figure 1B) and lung adenocarcinoma (Figure 1C). Ten STAS cases of CRC metastasis were analysed. The original tumours were diagnosed pathologically as well‐ or moderately differentiated adenocarcinoma (Figure 1D), and STAS polarity status was classified into four subtypes: complete apical‐out (Complete), partial apical‐out (Partial), mixed with apical‐in (Mix), and no polarity (None) (Figure 1E). Of the *n* = 55 CRC STAS regions in ten patients, 50.9% (28/55) were Complete, 21.8% (12/55) were Mix, 21.8% (12/55) were Partial, and 5.5% (3/55) were None. Thus, most examples of CRC STAS derived from well‐ or moderately differentiated adenocarcinoma showed apical‐out polarity. Of the *n* = 70 lung adenocarcinoma STAS regions in ten patients, 38.6% (27/70) were Complete, 0% (0/70) were Mix, 22.8% (16/70) were Partial, and 38.6% (27/70) were None (supplementary material, Figure S3C). Thus, the majority of STAS regions of adenocarcinoma of CRC and lung cancer showed apical‐out polarity, ranging from Complete to Mix.

### Cancer cell clusters have greater metastatic potential than do single cells in an *in vivo* STAS model

**Figure 2:**
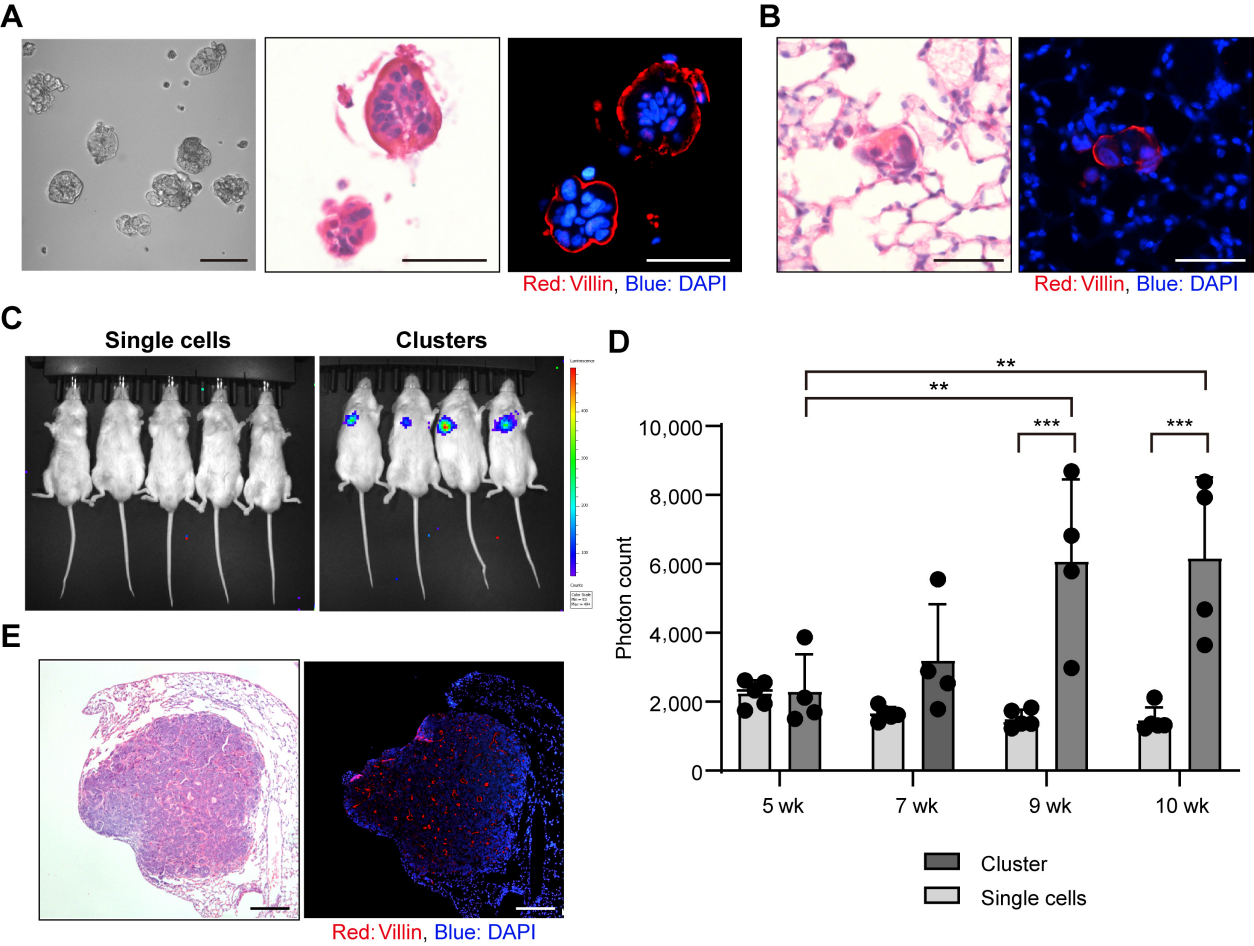
*In vivo* model of STAS in CRC lung metastases. (A) Polarity status of CRC clusters (C45 luc2) cultured in suspension. Left: brightfield. Scale bar: 100 μm. Centre: H&E staining. Right: immunostaining (red, villin; blue, DAPI). Scale bar: 50 μm. (B) Polarity status of a CRC cluster in the lung 1 day after intratracheal injection into a mouse. Left: H&E staining; right: immunostaining (red, villin; blue, DAPI). Scale bar 50 μm. (C) Bioluminescence imaging of mice 10 weeks after intratracheal injection of CRC clusters or single cells. Left: single cells injected group (*n* = 5); right: clusters injected group (*n* = 4). (D) Time course of bioluminescence intensity in mice at 5, 7, 9, and 10 weeks. **p < 0.01; ***p < 0.001. (E) Microscopic images of lung metastasis in the rightmost mouse in the cluster group (shown in panel C) 10 weeks after intratracheal injection of CRC clusters. Left: H&E staining; right: immunostaining (red, villin; blue, DAPI). Scale bar: 200 μm.

In CTCs, clusters of cancer cells have been reported to have greater metastatic potential than do single cells [11, 12]. To determine whether this is also true in metastasis through the air space of the lung, *n* = 1,000 cancer cell clusters or an equivalent number of single cells from C45/Luc2 organoids were intratracheally administered into mice. Similar to the polarity status of small clusters cultured in suspension (Figure 2A), small clusters in the lungs immediately after transplantation showed apical‐out status (Figure 2B). Metastases were monitored using bioluminescence imaging. The intensity increased with time only in cluster‐administered mice, but not in single cell‐administered mice (Figure 2C,D). Histopathological analysis showed that metastases were found only in the cluster‐administered mice, and the metastases showed apical‐in polarity (Figure 2E). Thus, clusters had a greater capacity for metastasis formation than did single cells, and polarity switching from apical‐out to apical‐in occurred in the process of forming metastatic foci.

### Establishment of an *in vitro* STAS model

We reported previously that polarity switching of cancer cell clusters plays an important role in metastasis in a mouse model of CRC liver metastasis [13]. To investigate the role of polarity switching of cancer cell clusters in STAS, an *in vitro* model in which CRC organoids and airway epithelium are co‐cultured was established. First, MAOs were prepared and cultured in 2D conditions (2D‐MAOs) according to reported protocols [16] (supplementary material, Figure S4A). 2D‐MAOs were found to differentiate into multiple cell types in the lung: acetylated tubulin^+^ ciliated cells, mucin 5AC^+^ goblet cells, CC10^+^ Clara cells, and caveolin^+^ type 1 alveolar cells (supplementary material, Figure S4B). Caveolin^+^ type 1 alveolar cells were predominant [18], indicating that 2D‐MAOs represent peripheral airway epithelium. To determine the culture medium for co‐culture, MAOs and organoids were cultured in mixed media at various ratios, and viability was evaluated after 7 days. MAOs cultured well in all of the mixing ratios (supplementary material, Figure S4C), but organoids showed a lower viability at higher ratios of MAO medium (supplementary material, Figure S4D). Therefore, the mixing ratio was set to 1:1.

**Figure 3:**
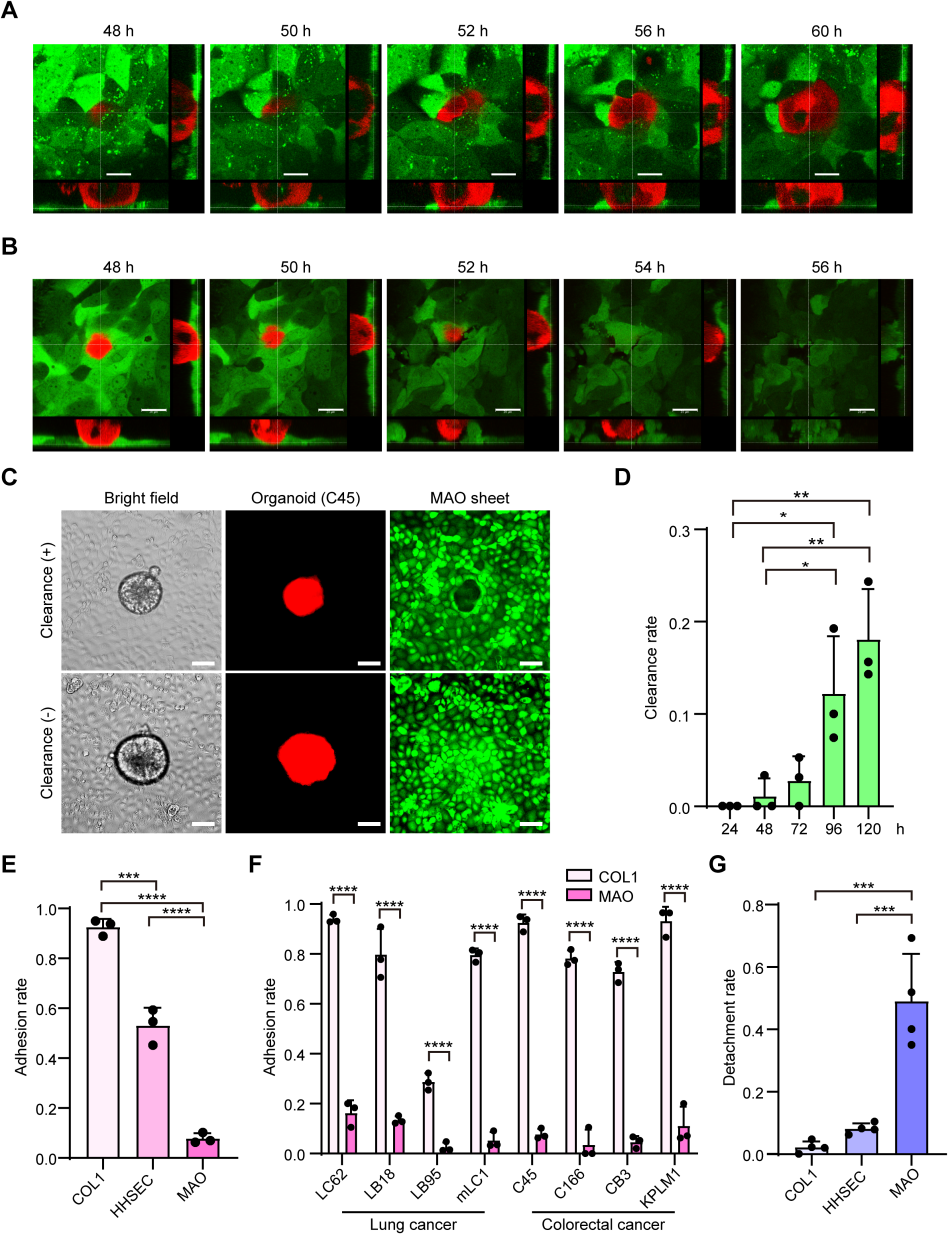
Attachment process of CRC organoids into mouse airway epithelial cell sheets. (A, B) Confocal imaging of CRC organoids and 2D‐MAOs over time. Red: C45 organoids labelled with mCherry; green: 2D‐MAO stained with CellTracker Green. CRC organoid infiltrating a 2D‐MAO and reaching the bottom of the culture dish (A) and CRC organoid detached after attaching to a 2D‐MAO (B). Scale bar: 20 μm. (C) Representative microscopic images of clearance: brightfield (left), mCherry (centre), and CellTracker Green (right). Representative images of clearance (top) and non‐clearance (bottom). A region with no fluorescence below an organoid, showing clearance (top right). Scale bar: 50 μm. (D) Clearance rates of C45 organoids at each time point after co‐culture. (E) Comparison of adhesion rates of C45 organoids to type I collagen (COL1), HHSECs, and 2D‐MAO. (F) Adhesion rates of other CRC and lung cancer organoids. Comparison of adhesion rates of type 1 collagen (COL1) and 2D‐MAO for each line is shown. (G) Comparison of detachment rates of C45 organoids to COL1, HHSECs, and 2D‐MAO. *p < 0.05; **p < 0.01; ***p < 0.001; ****p < 0.0001.

The interaction between CRC organoids and 2D‐MAOs was monitored over time by confocal microscopy. CRC organoids attached to 2D‐MAOs (adhesion), infiltrated into the intercellular spaces of MAO cells, attached to the dish, and expanded the area of attachment to the dish (clearance) by pushing away surrounding MAOs (Figure 3A and supplementary material, Figure S1). Interestingly, some of the organoids detached from the MAOs even after infiltrating into the intercellular spaces of the MAOs (detachment) (Figure 3B and supplementary material, Figure S1). The rate of clearance increased over time (Figure 3C,D). Adhesion of CRC organoids to 2D‐MAOs was significantly lower than adhesion to type 1 collagen‐coated dishes and HHSECs (Figure 3E). A lower ability to adhere to 2D‐MAOs than collagen‐coated dishes was observed in other CRC organoid lines, as well as lung cancer organoid lines (Figure 3F). The rate of detachment from 2D‐MAOs was significantly higher than the rate of detachment from type 1 collagen and HHSECs (Figure 3G). These results suggest that the airway epithelium plays a protective role against STAS‐derived intrapulmonary metastasis.

### Polarity status of CRC organoids is involved in adhesion to lung epithelial cells

**Figure 4:**
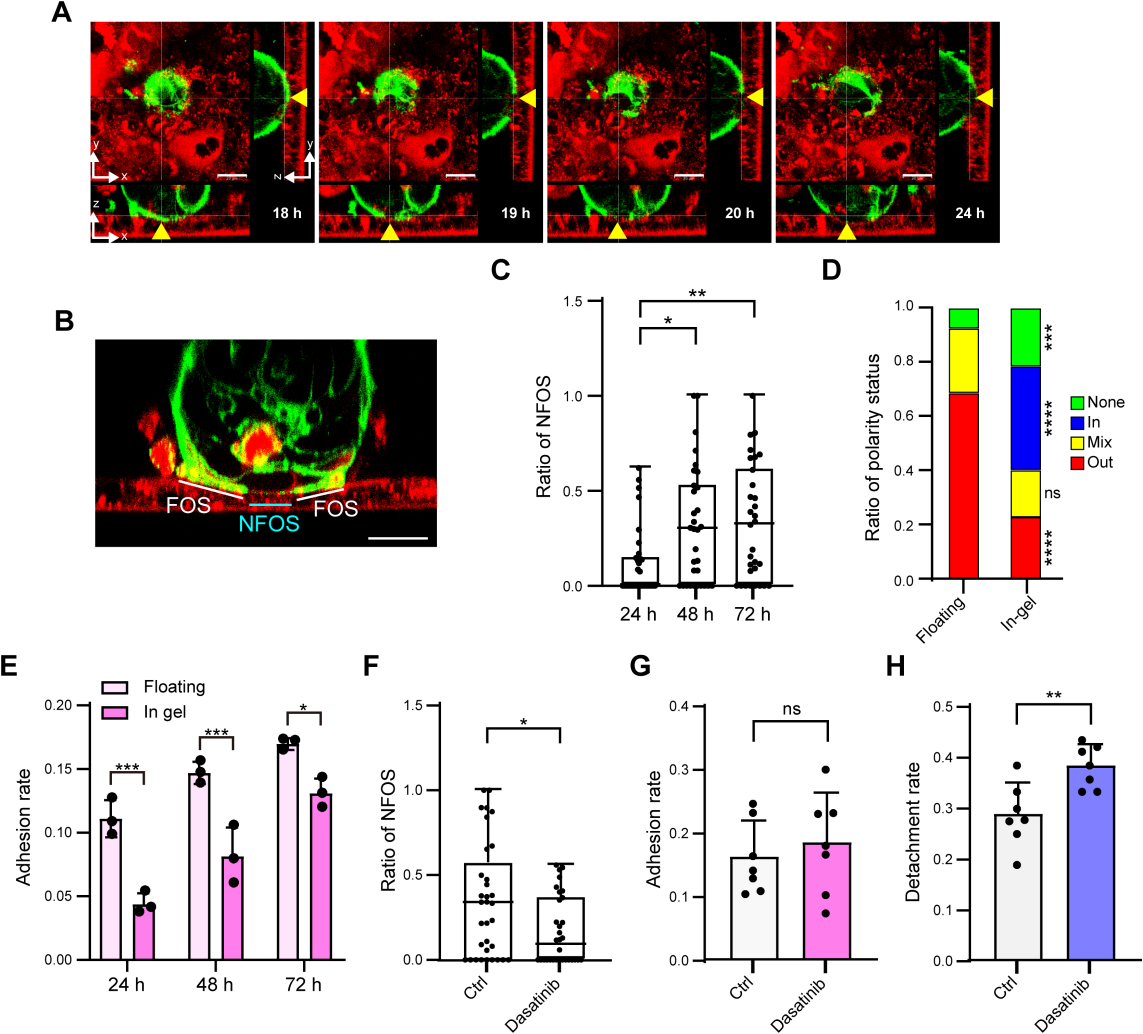
Dynamic polarity changes of CRC organoids upon adhesion to 2D‐MAOs. (A) Confocal images of CRC organoids and 2D‐MAOs at designated time points. Green: C45 organoids expressing GPI‐GFP; red: 2D‐MAO stained with CellTracker Red. Scale bar 20 μm. Regions that have no green fluorescence at the organoid surface (NFOS) are indicated by arrowheads. (B) Confocal images. Regions that have green fluorescence at the organoid surface (FOS) are indicated by yellow lines and NFOS by a blue line. Green: C45 organoids expressing GPI‐GFP; red: 2D‐MAO stained with CellTracker Red. Scale bar: 20 μm. (C) Quantitative analysis of the NFOS ratio over time. (D) Ratio of polarity status of organoids in the indicated conditions. In‐gel: 48 h in 5% Matrigel. (E) Comparison of the adhesion rates of organoids cultured in suspension (floating) and in Matrigel (in‐gel) at the designated time points. (F–H) NFOS ratios (F), adhesion rates (G), and detachment rates (H) with or without dasatinib treatment. ns, not significant; *p < 0.05; **p < 0.01; ***p < 0.001; ****p < 0.0001.

The dynamics of the apical membrane of an apical‐out organoid expressing GFP‐GPI reporter [14] in contact with 2D‐MAOs of co‐culture at 18 h were followed using confocal microscopy. Loss of the apical membrane already started to appear at 18 h, and the loss gradually expanded until 24 h (Figure 4A). The ratio of the apical marker of the organoids at the interface was quantified (Figure 4B). Apical membrane loss increased significantly from 24 to 48 h, reaching a plateau at 48 h (Figure 4C). Next, the role of the outer apical membrane of the organoids in adhesion to 2D‐MAOs was investigated. The polarity of CRC organoids changed depending on the culture conditions. When cultured in suspension, the apical membrane was predominantly located outside the organoids (apical‐out), and when cultured in gel, the apical membrane was predominantly located inside the organoids (apical‐in) [13] (Figure 4D). The rate of adhesion to 2D‐MAOs was higher for apical‐out than for apical‐in organoids at 24, 48, and 72 h (Figure 4E). In contrast, there was no difference in the adhesion rates after 24 h to type I collagen‐coated dishes between apical‐out and apical‐in organoids (supplementary material, Figure S5A). When polarity switching was inhibited by pre‐treatment with dasatinib, an SRC family kinase inhibitor [13], apical membrane loss decreased significantly at 48 h (Figure 4F), and there was no difference in the adhesion rate, but the detachment rate significantly increased (Figure 4G,H). These results indicate that the initial contact between the organoids and 2D‐MAOs allowed for initial adhesion, followed by the activation of apical–apical membrane sensor causing membrane remodelling at the contact site and establishing subsequent attachment. The importance of membrane remodelling for establishing attachment was also supported by the assay using C166 GFP‐GPI organoids derived from micropapillary carcinoma [14]. As shown in Figure 3F, the adhesion rate between MAOs and C166 was very low, with only nine adherence sites detected in this assay. Seven of these had completely apical‐out (NFOS = 0), and in the remaining two, the loss of GFP was incomplete (supplementary material, Figure S5B). The fact that C166 is less prone to polarity switching may be reflected in the difference in adhesion rates.

### TGF‐β1 promotes adhesion of CRC organoids to the airway epithelium

**Figure 5:**
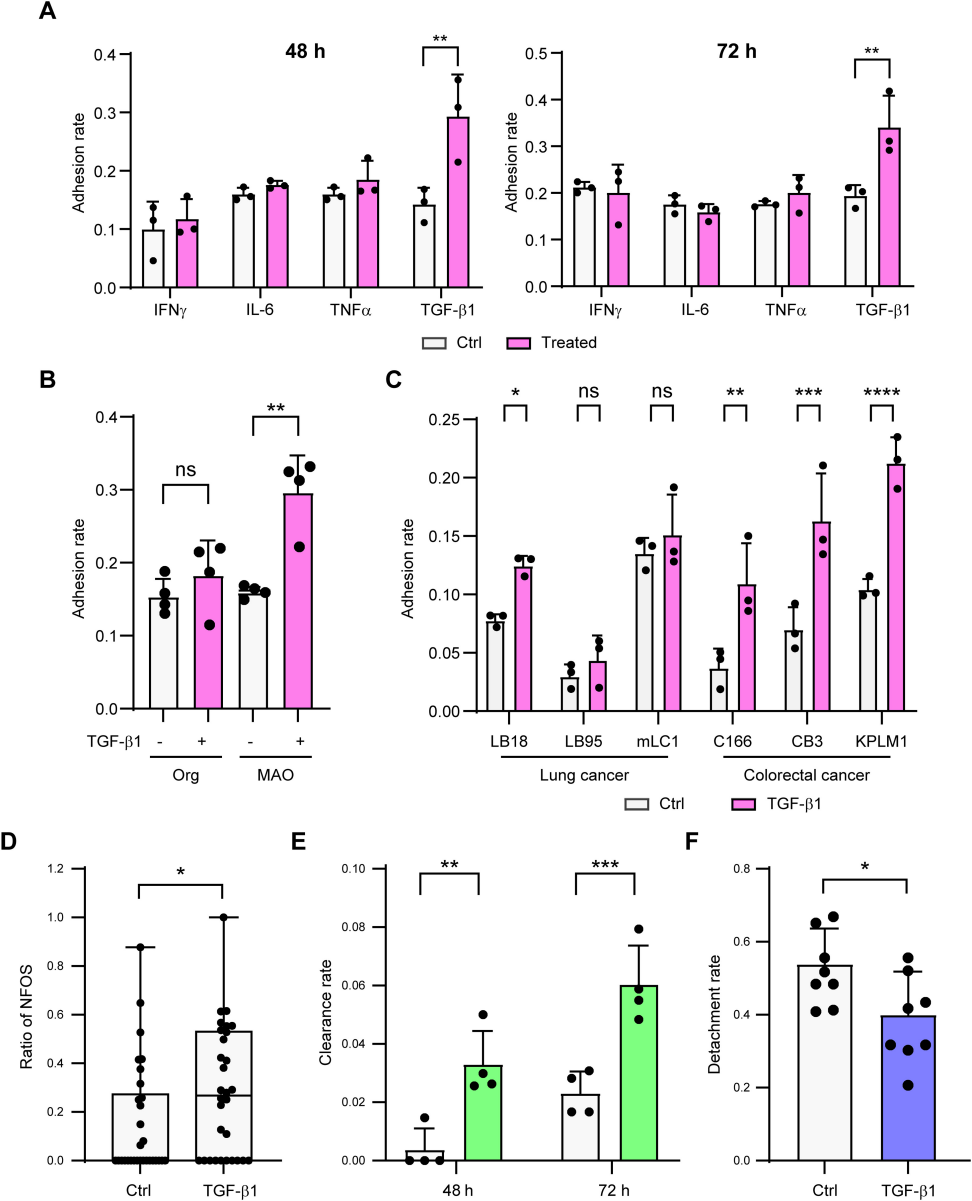
TGF‐β1‐treated 2D‐MAOs promote attachment of CRC organoids. (A) Adhesion rate of C45 organoids to 2D‐MAOs after 48 and 72 h of co‐culture in the left and right graphs, respectively. Both C45 organoids and 2D‐MAOs were pre‐treated with the indicated cytokines, and each cytokine was also added to the co‐culture medium. Control, H_2_O. (B) Adhesion rate of C45 organoids to 2D‐MAOs after 48 h co‐culture. Either C45 organoids or 2D‐MAOs were pre‐treated with TGF‐β1 before the assay. Org, organoids. (C) Adhesion rate of other lung cancer organoids and CRC organoids to 2D‐MAOs. 2D‐MAOs were pre‐treated with TGF‐β1 or H_2_O. (D–F) 2D‐MAOs were pre‐treated with TGF‐β1 or H_2_O. (D) Quantitative analysis of the no green fluorescence at the organoid surface (NFOS) ratio in C45/GPI‐GFP. (E) Clearance rate of C45 organoids at each time point. (F) Detachment rate of C45 organoids 24 h after the adhesion assay (shown in panel B). ns, not significant; *p < 0.05; **p < 0.01; ***p < 0.001; ****p < 0.0001.

Inflammation has been reported to promote lung metastasis [19, 20, 21]. In an *in vivo* model of intratracheal cancer cell administration, bleomycin‐induced inflammation promoted lung metastasis [22]. Thus, inflammation may promote intrapulmonary metastasis also due to STAS. Evaluation of the effect of inflammatory cytokines, IFN‐γ, IL‐6, TNF‐α, and TGF‐β1, on adhesion of C45 CRC organoids to 2D‐MAOs at a dose of 20 ng/ml found no effect on the viability of organoids or MAOs (supplementary material, Figure S6A). When TGF‐β1 was added, the adhesion rates of C45 CRC organoids to 2D‐MAOs were significantly increased at 48 and 72 h compared with the H_2_O control, whereas other cytokines had no effect on the adhesion rates (Figure 5A). To determine whether TGF‐β1 increased adhesion rates as a result of its action on either CRC organoids or MAOs, adhesion assays were performed after pre‐treatment of either. Pre‐treatment with TGF‐β1 prior to co‐culturing organoids and 2D‐MAOs increased the adhesion rates only with 2D‐MAO pre‐treatment, but not with CRC organoid pre‐treatment (Figure 5B and supplementary material, Figure S6B). Indeed, Smad3, downstream of TGF‐β1 signalling, was strongly phosphorylated in 2D‐MAOs by TGF‐β1 treatment (supplementary material, Figure S6C). Increased adhesion by TGF‐β1 pre‐treatment of 2D‐MAOs was observed in all four CRC organoids examined, including C45, and in one of the three lung cancer organoids (Figure 5B,C). When MAOs were pre‐treated with TGF‐β1, the ratios of NFOS of C45 CRC organoids and the clearance rate were increased (Figure 5D,E), and the detachment rate decreased (Figure 5F). These results suggest that airway epithelium exposed to TGF‐β1 promotes adhesion and invasion of the floating cancer cell clusters through accelerated loss of apical polarity.

### FSTL1 promotes CRC organoid adhesion downstream of TGF‐β1

**Figure 6:**
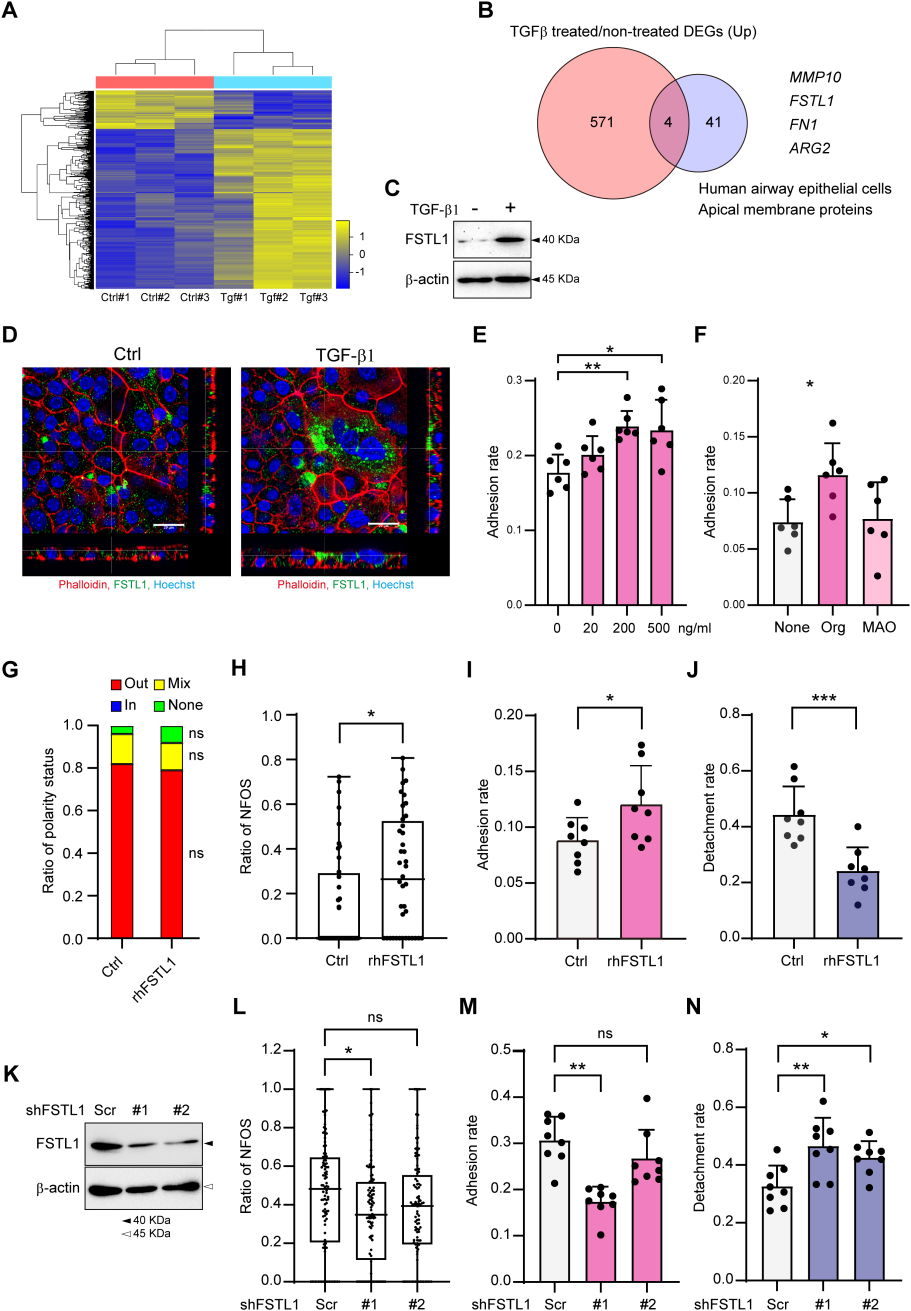
TGF‐β1‐induced FSTL1 secretion from 2D‐MAOs affects the attachment of CRC organoids. (A) Heatmap of 2D‐MAO microarray analysis. Triplicates for each condition (TGF‐β1‐treated, Tgf; non‐treated, Ctrl) are shown. (B) Venn diagram of differentially expressed genes (DEGs) upregulated in TGF‐β1‐treated 2D‐MAOs and listed genes in the database for apical membrane proteins in human airway epithelial cells. Four common genes are shown. (C) Western blotting for FSTL1 in 2D‐MAOs treated with TGF‐β1. (D) Immunostaining of FSTL1 for TGF‐β1‐treated 2D‐MAOs. Green, FSTL1; red, phalloidin; blue, Hoechst 33342. Scale bar: 20 μm (E) Adhesion rate of C45 organoids to 2D‐MAOs. The indicated doses of rhFSTL1 were added to the co‐culture medium. (F) Adhesion rate of C45 organoids to 2D‐MAOs. Either C45 (Org) or 2D‐MAOs (MAO) were pre‐treated with TGF‐β1 before the assay. (G) Ratio of polarity status of rhFSTL1‐treated (rhFSTL1) and non‐treated (Ctrl) organoids. (H–J) Quantitative analysis of the no green fluorescence at the organoid surface (NFOS) ratio (H), adhesion rate (I), and detachment rate (J) for rhFSTL1‐treated (rhFSTL1) and non‐treated (Ctrl) organoids. (K) Western blotting of FSFL1 infected with two different shRNAs against FSTL1 (#1 and #2) and shRNA with scrambled sequence (Scr). (L–N) Quantitative analysis of NFOS ratio (L), adhesion rate (M), and detachment rate (N) for shFSTL1‐infected (#1, #2) and scramble shRNA‐infected (Scr) organoids. Organoids were treated with TGF‐β1 (K–N). ns, not significant; *p < 0.05; **p < 0.01; ***p < 0.001.

To examine the downstream effect of TGF‐β1 in 2D‐MAOs promoting CRC organoid adhesion, gene expression profiles were analysed and compared between TGF‐β1‐treated and non‐treated 2D‐MAOs (Figure 6A). The number of differentially expressed genes (DEGs) that showed significantly more than two‐fold or greater changes was *n* = 709, with *n* = 571 upregulated DEGs and *n* = 138 downregulated DEGs (Figure 6B). Gene set enrichment analysis (GSEA) showed that the signatures of TGF‐β1 signalling and epithelial–mesenchymal transition (EMT) were enriched in TGF‐β1‐treated MAOs (supplementary material, Figure S7). Since adhesion of CRC organoids and MAOs occurs at the apical membrane of both, apical membrane proteins were further examined. Four of the DEGs were included in the PolarProtDb database [23] of lung epithelial apical membrane proteins (Figure 6B). The expression level of the top DEG, matrix metalloproteinase 10 (*MMP10*), was low, so the second gene, follistatin‐like protein 1 (*FSTL1*), was examined. FSTL1 is a secreted glycoprotein belonging to the SPARC (secreted protein acidic and rich in cysteine) family, which modulates cell interactions with the extracellular environment [24]. Several studies have shown that FSTL1 is involved in systemic autoimmune disease, developmental processes, cardiovascular diseases, and cancer [25]. Western blotting results showed that FSTL1 levels were low in non‐treated MAOs, but they increased with TGF‐β1 treatment (Figure 6C). FSTL1 induced by TGF‐β1 was confirmed to be localised to the apical membranes of 2D‐MAOs (Figure 6D). Next, the functional role of FSTL1 in the adhesion of CRC organoids to 2D‐MAOs was examined using rhFSTL1. The cell viability of C45 organoids and 2D‐MAOs was not affected by rhFSTL1 up to 1,000 ng/ml (supplementary material, Figure S8A). When rhFSTL1 was added to the co‐culture medium, the adhesion rates increased dose‐dependently (Figure 6E). Pre‐treatment with rhFSTL1 prior to co‐culturing organoids and 2D‐MAOs increased the adhesion rates only with organoid pre‐treatment, but not with 2D‐MAO pre‐treatment (Figure 6F). These results suggest that TGF‐β1‐induced secretion of FSTL1 may act on CRC organoids to promote adhesion. Next, the molecular mechanisms by which adhesion is promoted by FSTL1 in C45 organoids were investigated. FSTL1 has been reported to activate SRC family kinases (SFKs) in CRC cell lines [26]. Furthermore, activation of SFK plays an important role in the polarity switching of CRC organoids [13]. Although rhFSTL1 stimulated the phosphorylation of SFK (supplementary material, Figure S8B), polarity switching was not induced by rhFSTL1 alone (Figure 6G). Co‐culture of rhFSTL‐pre‐treated C45 organoids and 2D‐MAOs significantly increased the NFOS ratio (Figure 6H) and adhesion rates (Figure 6I) and decreased the detachment rates (Figure 6J). Finally, *FSTL1* gene expression was suppressed by shRNA in MAOs. The levels of FSTL1 protein induced by TGF‐β1 were suppressed by two shFSTL1 constructs (Figure 6K). Co‐culture of C45 organoids and 2D‐MAO/shFSTL1#1 pre‐treated with TGF‐β1 significantly decreased the NFOS ratio (Figure 6L) and adhesion rates (Figure 6M) and increased the detachment rates (Figure 6N). There was also a significant increase in the detachment rates of C45 organoids from 2D‐MAO/shFSTL1#2 (Figure 6N), but the decreases in NFOS and the adhesion rate were not significant (Figure 6L,M). FSTL1 may play a role mainly in the detachment step. These results suggest that FSTL1 secreted from the lung epithelium by the pro‐inflammatory cytokine TGF‐β1 may promote STAS adhesion to the lung epithelium through SFK activation of CRC STAS and accelerated apical membrane loss at the adhesion interface. Although the effects of FSTL1 knockdown were not drastic, FSTL1 secretion may be one of the mechanisms by which TGF‐β1 stimulates cancer cluster attachment to the airway epithelium.

## Discussion

An *in vitro* STAS model was successfully established using co‐culture of cancer organoids with airway epithelium. Using this model, dynamic changes in the polarity of cancer cell clusters were experimentally shown at the interface of adhesion to the airway epithelium. Furthermore, cancer cell adhesion in the lung was enhanced by the preceding TGF‐β1 treatment of the airway epithelium, which could suggest the crucial role of locoregional inflammation in STAS‐pulmonary metastasis formation.

The involvement of polarity switching in the invasion of cancer cell clusters has been reported in liver metastases of CRC [13] and peritoneal metastases of ovarian cancer [15]. The region of the cancer cell mass exposed to free space forms the apical membrane, and cancer cell clusters exhibit the apical‐out status in floating conditions. Early in the invasive process, floating cancer cell clusters with apical‐out status lose apical membrane after adhesion to the airway epithelium and are eventually converted to apical‐in status [13, 15], although it may depend on the genetics of the cells and the type of environment the clusters are invading [27]. This study showed that these events also occur during invasion of CRC and lung cancer clusters into the airway epithelium. Furthermore, the airway epithelium played a protective role against the adhesion of CRC cell clusters. This is consistent with the well‐known protective function of the airway epithelium against foreign substances [28]. Compared with type I collagen‐coated culture dishes, the adhesion capacity of cancer cell clusters to culture dishes confluently covered with MAOs was greatly diminished. This may be due to negative charges in the apical membrane preventing their first contact. The subsequent detachment of some of the previously attached tumour cell clusters suggests an active protective function of the airway epithelium. If the epithelium is damaged, and the stroma is exposed to the airway lumen, there may be an increased risk of adhesion of the tumour cell mass. Avoiding damage to the airway epithelium may prevent recurrence due to STAS.

In the present study, FSTL1 secreted from TGF‐β1‐stimulated airway epithelium exhibited a paracrine action on CRC organoids to promote attachment. The role of FSTL1 in cancer is complex because of the diversity of FSTL1‐producing cells, receptors, and intracellular signals [29]. Several molecules have been reported in FSTL1‐mediated signalling, including bone morphogenetic protein (BMP) receptors [29], disco interacting protein 2 homolog A (DIP2A) [30], and vimentin (VIM) [26]. The growth of C45 CRC organoids is inhibited by the BMP receptor inhibitor LDN193189 [31], but no growth‐inhibitory effect by rhFSTL1 was observed in the present study (supplementary material, Figure S7A), suggesting that adhesion promotion by rhFSTL1 may not be mediated by BMP signalling. On the other hand, in CRC cell lines (DLD1, RKO), FSTL1 has been reported to phosphorylate FAK, paxillin, and SRC via binding to VIM [26]. Since SFK was phosphorylated also by rhFSTL1 in C45 (supplementary material, Figure S7C), and inhibition of Src phosphorylation by dasatinib suppressed polarity switching and adhesion to MAOs, it is possible that the signal was transmitted to C45 via VIM. However, the expression levels of VIM and DIP2A in C45 are quite low and may be mediated by other unknown receptors. Since the effect of knockdown of FSTL1 was not drastic, there may be other mechanisms by which TGF‐β1 promotes attachment than by stimulating FSTL1 secretion. GSEA showed that EMT is activated in TGF‐β1‐stimulated MAOs; it is also possible that EMT is involved in enhanced attachment of cancer cell clusters to MAOs. EMT may weaken intercellular contacts between airway epithelia and allow CRC organoid infiltration.

Although the correlation with a poor prognosis supports the existence of STAS, some argue that STAS is an artefact of surgical manipulation or pathology specimen preparation [9]. However, because STAS showed complete apical‐out polarity in many patients’ tumours and polarity switch from apical‐in to apical‐out took several hours, artefact alone cannot explain the results of the present study. In contrast, the part of the main tumour facing the air space was apical‐out, so artefact cannot be ruled out when the cancer cell clusters show partial apical‐out.

As for whether cancer cell clusters can survive in the airway, it is impossible for them to remain suspended in the air at all times, given gravity. Long‐term survival in the alveoli is not always necessary, since clearance of the alveolar epithelium begins at about 48 h (Figure 3D). A short period of suspension in the alveoli at near 100% humidity would not affect the survival of cancer cell clusters. In addition, the air–liquid interface method, an organoid culture method [32], also suggests that a portion of the cancer cell cluster contacting the alveolar epithelium, which is covered with epithelial lining fluid, is sufficient for survival.

In this study, an *in vitro* model of STAS was established, and the adhesion of cancer cell clusters to the airway epithelium was examined. We have shown in several experiments that there is a correlation between the loss of the apical membrane of CRC clusters and their adhesion to the airway epithelium. The timing of the two events coincided, the adhesion was suppressed by a compound or proteins that blocked the polarity change, and organoids from micropapillary carcinomas in a stable apical‐out state had low adhesion rates. Unfortunately, there is no genetic manipulation to make CRC organoids permanently apical‐out, but these findings strongly suggest a correlation between apical membrane loss and adhesion. In addition, this model may be too simplistic to assess the entire process of STAS formation and invasion. For example, the mechanism by which cancer cell clusters are released into the air space remains to be elucidated. It is quite possible that weakened intercellular adhesion of cancer cells is involved in the development of STAS. Furthermore, mechanical forces generated by respiratory movements, coughing, surgical manipulation, etc. may facilitate the release of cancer cell clusters. Air–liquid interface cultures [33] or *ex vivo* lung slices [34] may help to assess more complex processes *in vivo*. Thus, clarifying the various processes including polarity switching in STAS could contribute to the development of therapies for patients with pulmonary malignancies associated with STAS.

## Author contributions statement

YM contributed to formal analysis, investigation, methodology, visualisation and writing of the original draft. KO contributed to data curation, formal analysis, supervision and writing – review and editing. RC contributed to methodology, supervision and writing – review and editing. HU contributed to formal analysis and investigation. JK contributed to supervision and writing – review and editing. AM‐H and NT‐M contributed to formal analysis and writing– review and editing. KK, TF and SO contributed to resources and writing – review and editing. MS and MyI contributed to resources, supervision and writing – review and editing. MhI contributed to conceptualisation, formal analysis, funding acquisition, project administration, supervision, visualisation and writing of the original draft.

## Supporting information


Supplementary materials and methods
**Figure S1.** Schematic diagram of the attachment process of cancer organoids to 2D‐MAOs
**Figure S2.** Adhesion and detachment assay protocols for cytokine, TGF‐β1, dasatinib, and rhFSTL1 treatment
**Figure S3.** Polarity status of colorectal cancer lung metastasis and lung cancer STAS
**Figure S4.** Characterisation of mouse airway organoids
**Figure S5.** Polarity status of CRC organoids and their adhesion to type I collagen or MAOs
**Figure S6.** Effect of cytokines on the viability and adhesion rates of CRC organoids and 2D‐MAOs
**Figure S7.** Gene set enrichment analysis (GSEA) between TGF‐β1‐treated and non‐treated 2D‐MAOs
**Figure S8.** Effect of rhFSTL1 on viability and SFK activation
**Table S1.** List of the reagents used

## Data Availability

Microarray data have been deposited in NCBI's Gene Expression Omnibus (GEO) and are accessible through GEO Series accession number GSE255842 (https://www.ncbi.nlm.nih.gov/geo/query/acc.cgi?acc=GSE255842). The data underlying all findings of this study are available from the corresponding author upon reasonable request. The data that support the findings of this study are openly available in GEO, reference number GSE255842.
