# Supplementary material for: Dynamic change of polarity in spread through air spaces of pulmonary malignancies
Source: J Pathol. 2025 Jan 13;265(3):260–73. doi: 10.1002/path.6382 (PMC11794978; doi:10.1002/path.6382)
Supplement: Supplementary file 1 — Supplementary materials and methods Figure S1. Schematic diagram of the attachment process of cancer organoids to 2D‐MAOs Figure S2. Adhesion and detachment assay protocols for cytokine, TGF‐β1, dasatinib, and rhFSTL1 treatment Figure S3. Polarity status of colorectal cancer lung metastasis and lung cancer STAS Figure S4. Characterisation of mouse airway organoids Figure S5. Polarity status of CRC organoids and their adhesion to type I collagen or MAOs Figure S6. Effect of cytokines on the viability and adhesion rates of CRC organoids and 2D‐MAOs Figure S7. Gene set enrichment analysis (GSEA) between TGF‐β1‐treated and non‐treated 2D‐MAOs Figure S8. Effect of rhFSTL1 on viability and SFK activation Table S1. List of the reagents used [file PATH-265-260-s001.docx]

**Dynamic change of polarity in spread through air spaces of pulmonary malignancies**

Y Matsuura *et al. J Pathol* <https://doi.org/10.1002/path.6382>

**Supplementary materials and methods**

**Supplementary Figures S1–S8**

**Supplementary Table S1**

**Supplementary materials and methods**

Reference numbers refer to the main text list.

*Ethics statement*

The study was approved by the Institutional Ethics Committees at Kyoto University (R1575, R2444), Kyoto Prefectural University of Medicine (ERB-C-1807), and Osaka International Cancer Institute (1803125402). Fresh surgical samples from patients were obtained with the patients’ informed consent. The animal studies were approved by the Institutional Animal Care and Use Committee of Kyoto University (18564).

*Confocal imaging for assessing interactions between CRC organoids and MAOs*

Dissociated MAOs were suspended in 2 ml of MAO medium and plated in glass-bottom dishes (Matsunami, Osaka, Japan; D11530H) coated with Cellmatrix Type IA at 1.5 × 10^6^ cells per dish. The medium was changed on day 3. Before the adhesion assay on day 7, 2D-MAOs were stained with CellTracker Green or Red for 1 h and washed twice with PBS. MAOs and *n* = 1,000 CRC organoids were co-cultured in co-culture medium as detailed below. After 48 h, the plates were washed with PBS twice, and 2 ml of co-culture medium was added. Time-lapse confocal images were acquired using a Leica TCS SPE confocal microscope (Leica Microsystems, Wetzlar, Germany). The bottom position was set 2–3 µm below the MAOs, and the top position was set at 24 µm (25 slices) and 39.5 µm (80 slices) from the bottom position for mCherry-organoid/green-MAOs and GPI-GFP-organoid/red-MAOs, respectively. Reconstruction of the longitudinal images in the *xz* and *yz* directions was performed using Leica Application Suite X software (version 3.5.7.23225).

*Cell preparation and culture*

Cancer organoids were prepared from patient tumour samples or xenografts and cultured as previously described [35, 36]. For preparation of apical-out organoids, organoids were cultured in suspension. For apical-in organoids, organoids were embedded in 5% Matrigel for 48 h. To avoid Matrigel effects, both types of organoids were treated with type 4 collagenase prior to the following assays. MAOs were prepared according to the previously reported protocol [16]. For 2D-MAOs, organoids were dissociated into single cells by incubation with TrypLE Express (Thermo Fisher Scientific, Waltham, MA, USA) for 10 min and plated on Cellmatrix Type IA (Nitta Gelatin Inc., Osaka, Japan)-coated wells. The medium was changed on day 3, and the 2D-MAOs became confluent on day 7. Primary human hepatic sinusoidal endothelial cells (HHSECs) isolated from human liver were purchased from ScienCell Research Laboratories (Carlsbad, CA, USA; 5000) and cultured in endothelial cell medium (ECM; ScienCell Research Laboratories; 1001) in bovine plasma fibronectin (ScienCell Research Laboratories; 8284)-coated culture dishes.

*Cell viability assay*

Organoids of similar size and shape (diameter 40–100 μm) were collected and seeded in non-treated, 24-well plates at a density of 100 organoids per well. Organoids were cultured for 1 week in the cancer organoid medium containing the indicated dose of the reagents (*n* = 3 wells for each condition). Pictures of the entire well were captured on day 0. The viabilities of the organoids were evaluated using the CellTiter-Glo assay (Promega, Madison, WI, USA; G7570), and the chemiluminescence value was obtained using a GloMax Discover Microplate Reader (Promega). Relative ATP values were calculated by dividing the chemiluminescence value on day 7 by the area of the organoids on day 0.

*Plasmids*

mCherry was introduced to C45 CRC organoids by lentiviral infection using pRRL-SV40-mCherry [37]. The target sequences of shRNA for FSTL1 were as follows: shFSTL1 #1, 5'-AGGCAGTAACTACAGTGAGAT-3'; shFSTL1 #2, 5'-GCAGAATGAAACAGCCATCAA-3' using pLKO.1-TRC cloning vector. Lentiviral infection was performed as previously described [15].

*Microarray analysis*

2D-MAOs were plated on six-well plates as above and treated with or without TGF-β1 in medium without noggin and A83-01 on day 3. The cells were collected on day 7. Total RNA was extracted as previously described. Microarray analysis, from three biological replicates, was performed at Macrogen Japan (Tokyo, Japan) using SurePrint G3 Mouse Gene Expression 8×60K (Agilent, Inc., Santa Clara, CA, USA). Labelled cRNAs were prepared from 1–5 µg of total RNA using Agilent’s Quick Amp Labeling Kit. Following fragmentation, 1.65 µg of cRNA was hybridised to the Agilent expression microarray following the manufacturer’s protocol. Arrays were scanned using the Agilent Technologies G4900DA SG12494263. Array data export processing and analysis were performed using Agilent Feature Extraction v11.0.1.1. A matrix of log_2_ signal values was normalised using a normalisation based on quantiles. A heatmap was created using Morpheus (<https://software.broadinstitute.org/morpheus>). For gene set enrichment analysis (GSEA), software was downloaded from the GSEA website (http://www.broad.mit.edu/gsea/downloads.jsp). GSEA was performed using the HALLMARK_EPITHELIAL_MESENCHYMAL_TRANSITION (MM3889) and the HALLMARK_TGF-Β1 _BETA_SIGNALING (MM3865) mouse gene sets [38] to identify enriched or depleted signatures. Gene sets with a false discovery rate (FDR) < 0.25 and a nominal *p* value < 0.05 were considered significant.

*Western blotting*

Western blotting analyses were performed as previously described [15] using the antibodies listed in supplementary material, Table S1. All antibodies were used at the manufacturer’s suggested concentrations. For the TGF-β1 treatment experiments, 2D-MAOs were treated with 20 ng/ml TGF-β1, cultured for 4 days, and subjected to analysis. For the rhFSTL1 treatment experiments, C45 organoids were treated with the indicated doses of rhFSTL1, cultured for 24 h, and subjected to analysis.

**Supplementary Figures S1–S8**

**
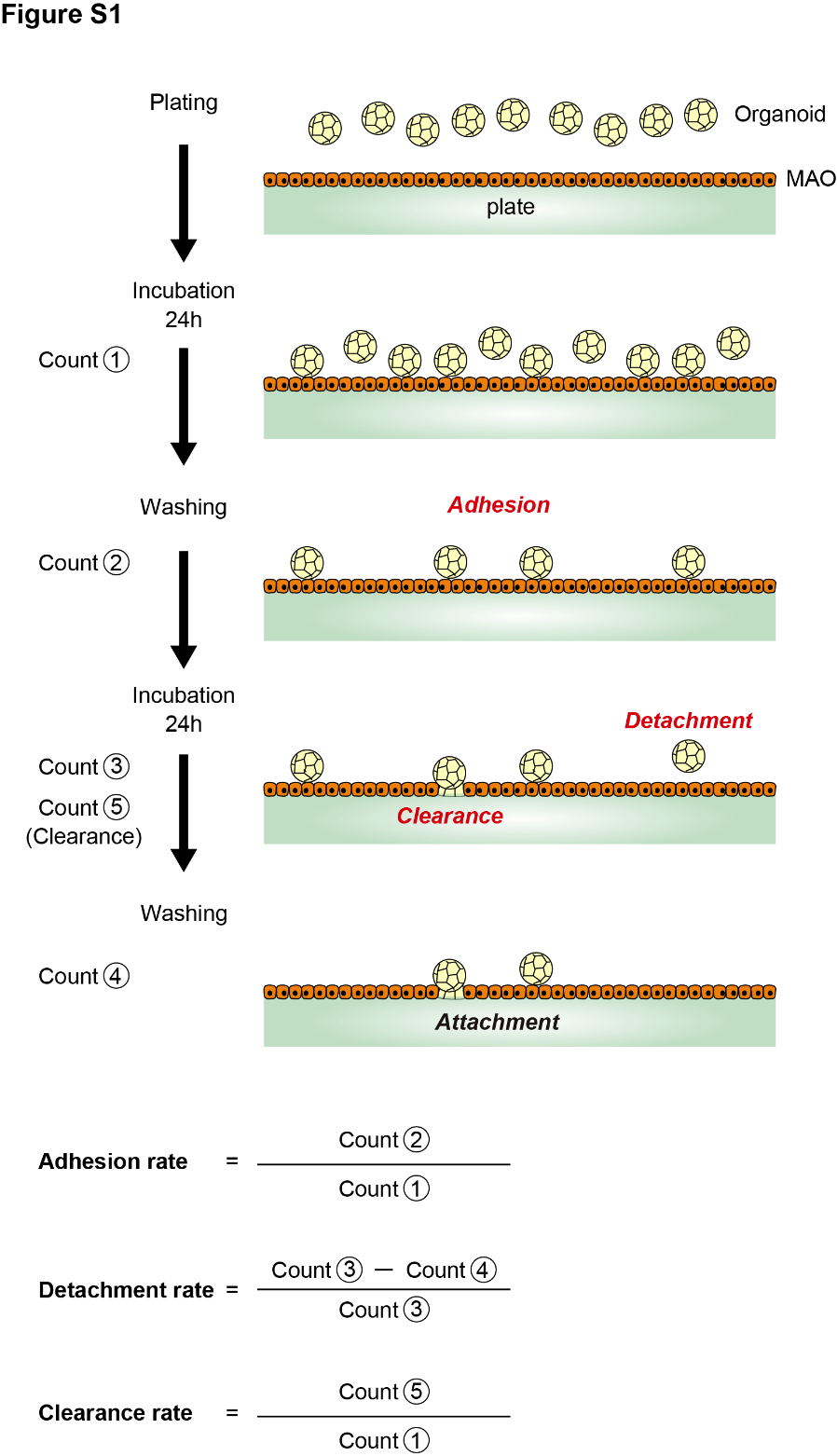
**

**Figure S1.** Schematic diagram of the attachment process of cancer organoids to 2D-MAOs. Functional items analysed are indicated in red.

**
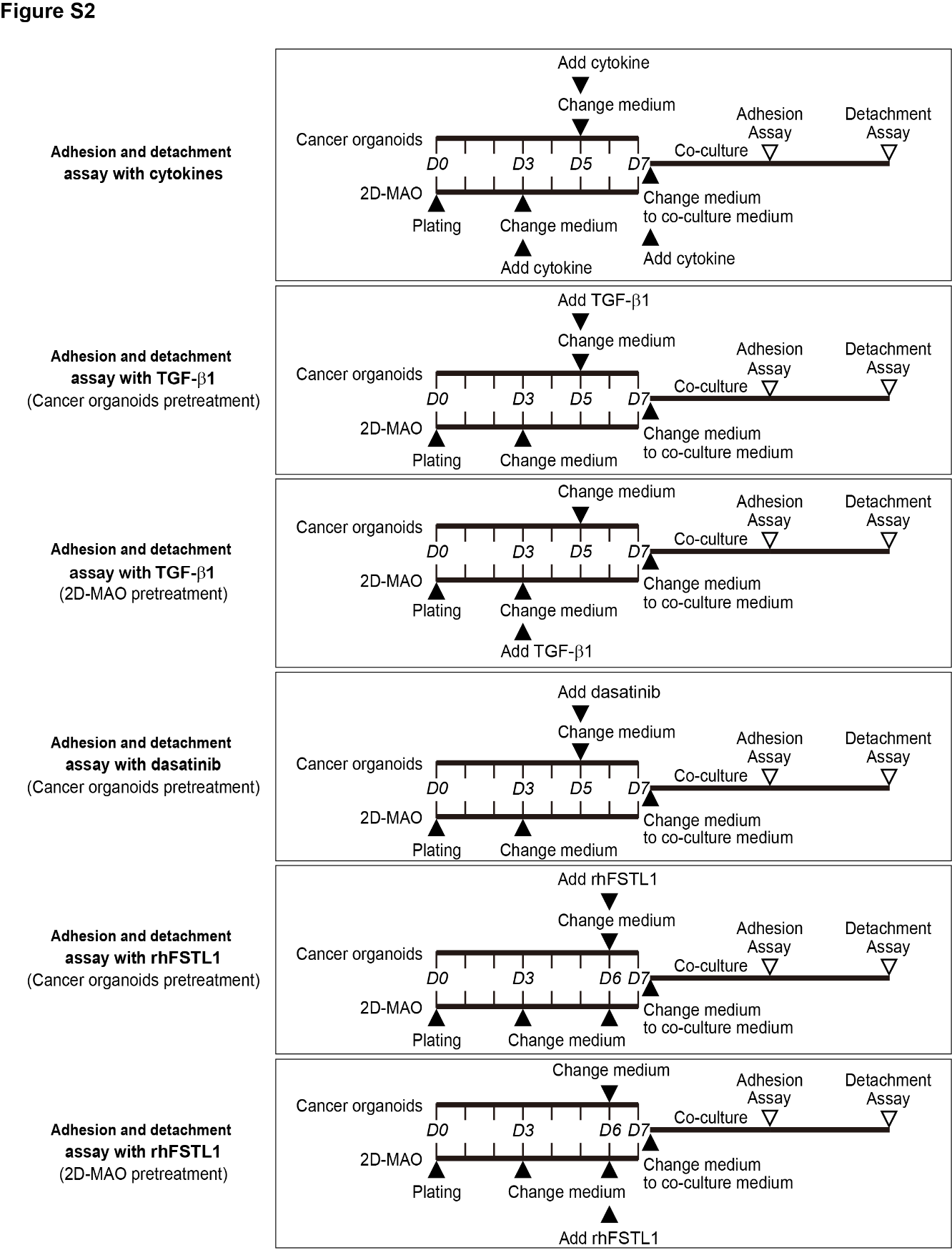
**

**Figure S2.** Adhesion and detachment assay protocols for cytokine, TGF-β1, dasatinib, and rhFSTL1 treatment.

**
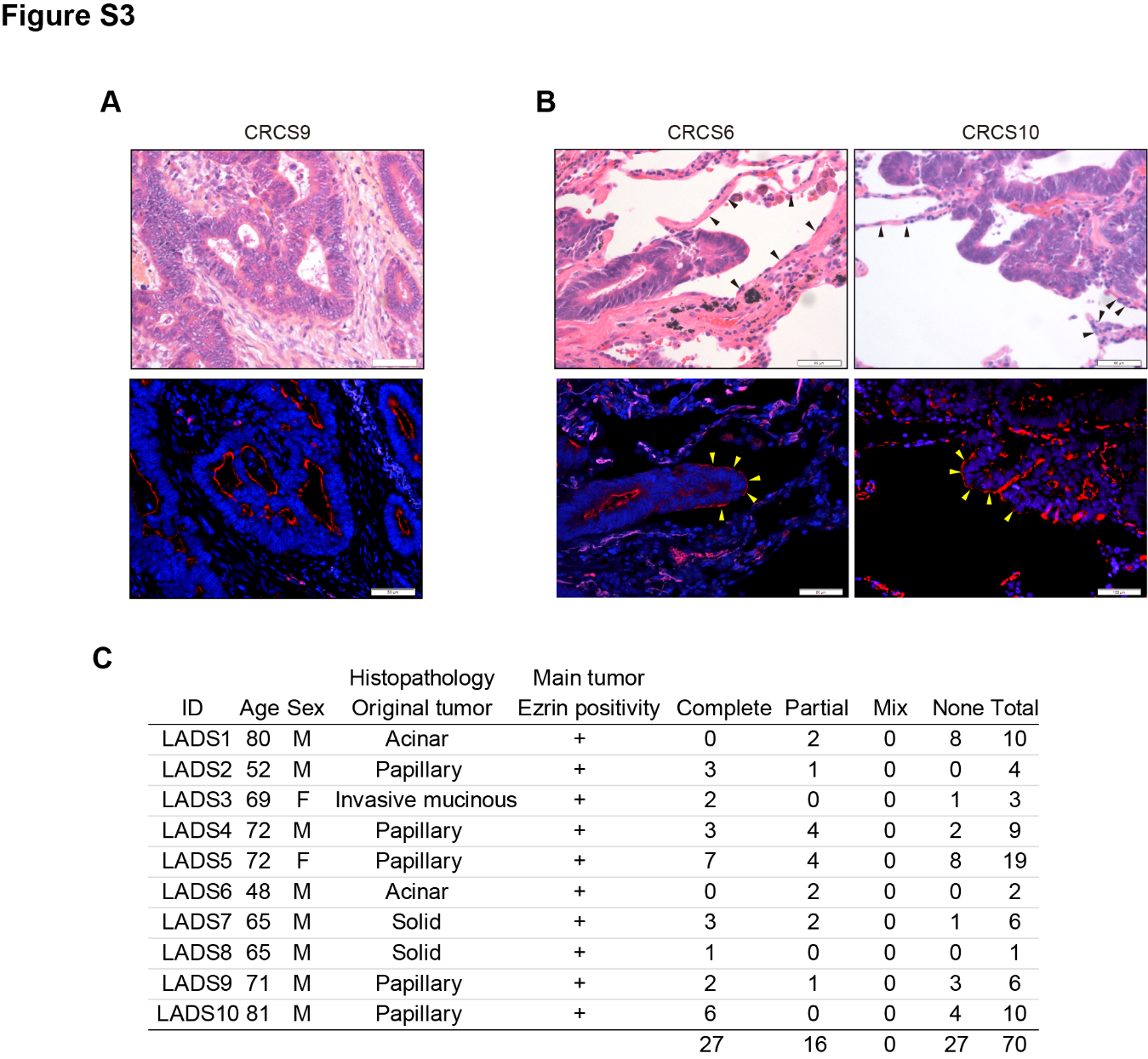
**

**Figure S3.** Polarity status of colorectal cancer lung metastasis and lung cancer STAS. (A) Main region of CRC lung metastasis in case CRCS9. Haematoxylin and eosin (H&E) staining and immunostaining. Red, villin; blue, DAPI. Scale bar: 50 μm. (B) Main region of CRC lung metastasis with exposed portion in the air space in cases CRCS6 and CRCS10. H&E staining (top) and immunostaining (bottom). Red, villin; blue, DAPI. Scale bar: 50 μm. The alveolar epithelium and the portion of tumour exposed in the air space are indicated by black and yellow arrowheads, respectively. (C) Summary of polarity status of the STAS regions in intrapulmonary metastases of lung adenocarcinomas.


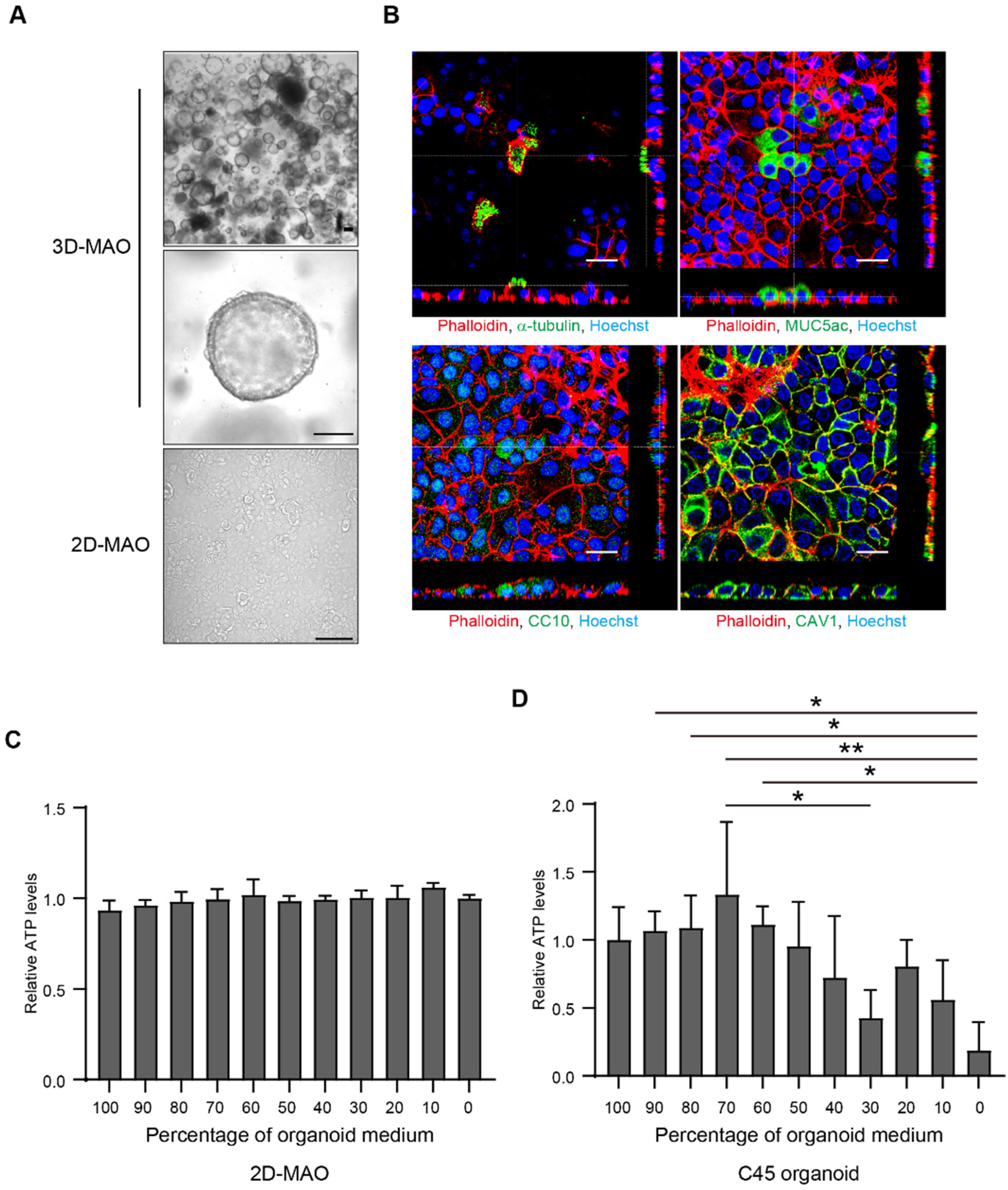


**Figure S4.** Characterisation of mouse airway organoids. (A) Brightfield images of mouse airway organoids (MAOs) cultured in 3D (3D-MAOs) and 2D (2D-MAOs). Scale bar: 100 μm. (B) Immunofluorescence staining of 2D-MAOs against differentiation markers in lung epithelium. Green: acetylated α-tubulin (left, top), MUC5AC (right, top), CC10 (left, bottom), and caveolin-1 (right, bottom); red: phalloidin; blue: Hoechst 33342. Scale bar: 20 μm. (C, D) Cell viability of 2D-MAOs (C) and C45 organoids (D) in co-culture medium, with the percentage of StemPro medium shown on the *X*-axis. **p*< 0.05; ***p*< 0.01.


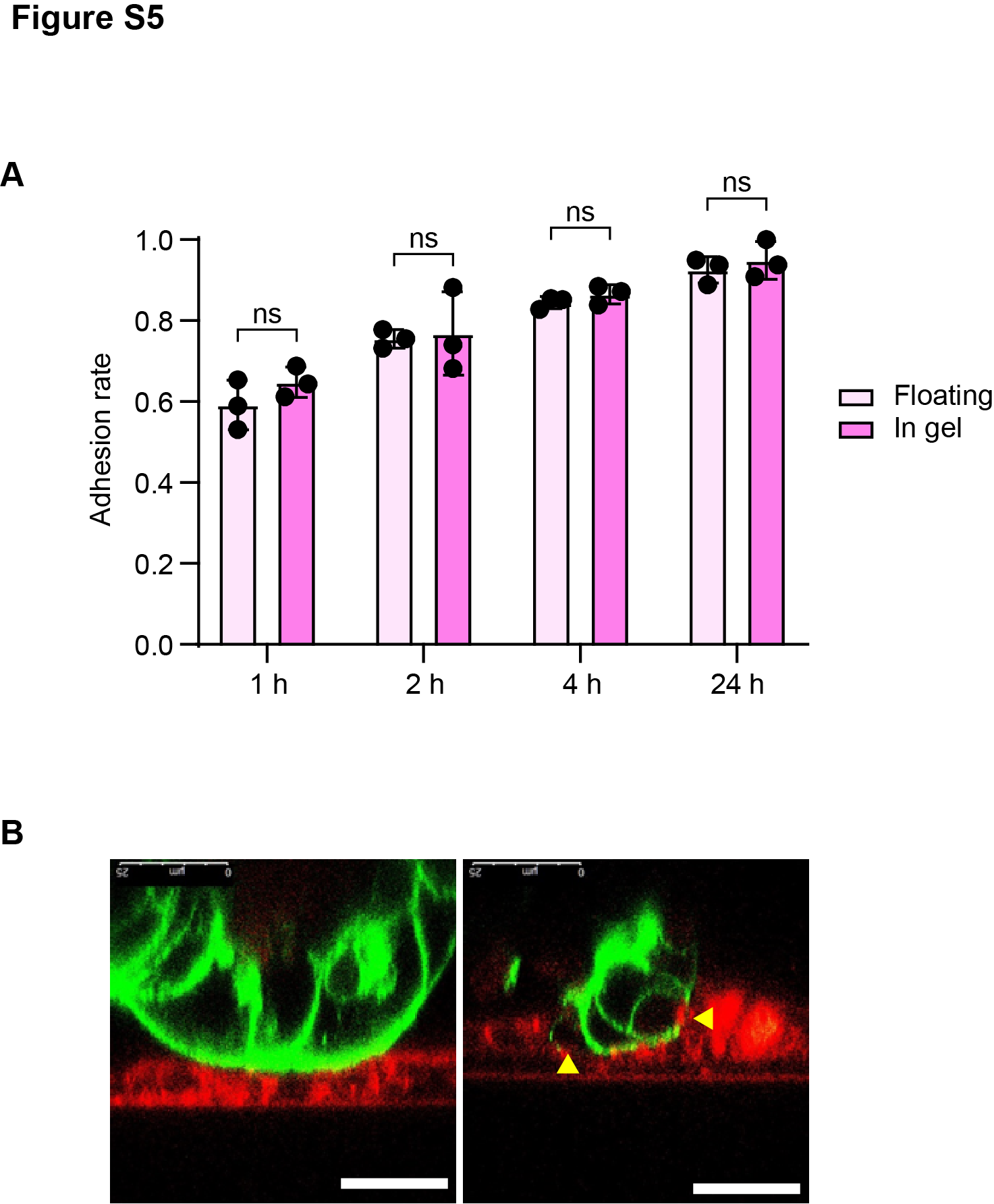


**Figure S5.** Polarity status of CRC organoids and their adhesion to type I collagen or MAO. (A) Comparison of the adhesion rates of organoids cultured in suspension (floating) and in 5% Matrigel (in-gel) to type I collagen-coated dishes at the designated time points. (B) Confocal images of CRC organoids and 2D-MAOs at 48 h. Green: C166 organoids expressing GPI-GFP; red: 2D-MAO stained with CellTracker Red. Scale bar: 25 μm. Regions that have no green fluorescence at the organoid surface (NFOS) are indicated by arrowheads.


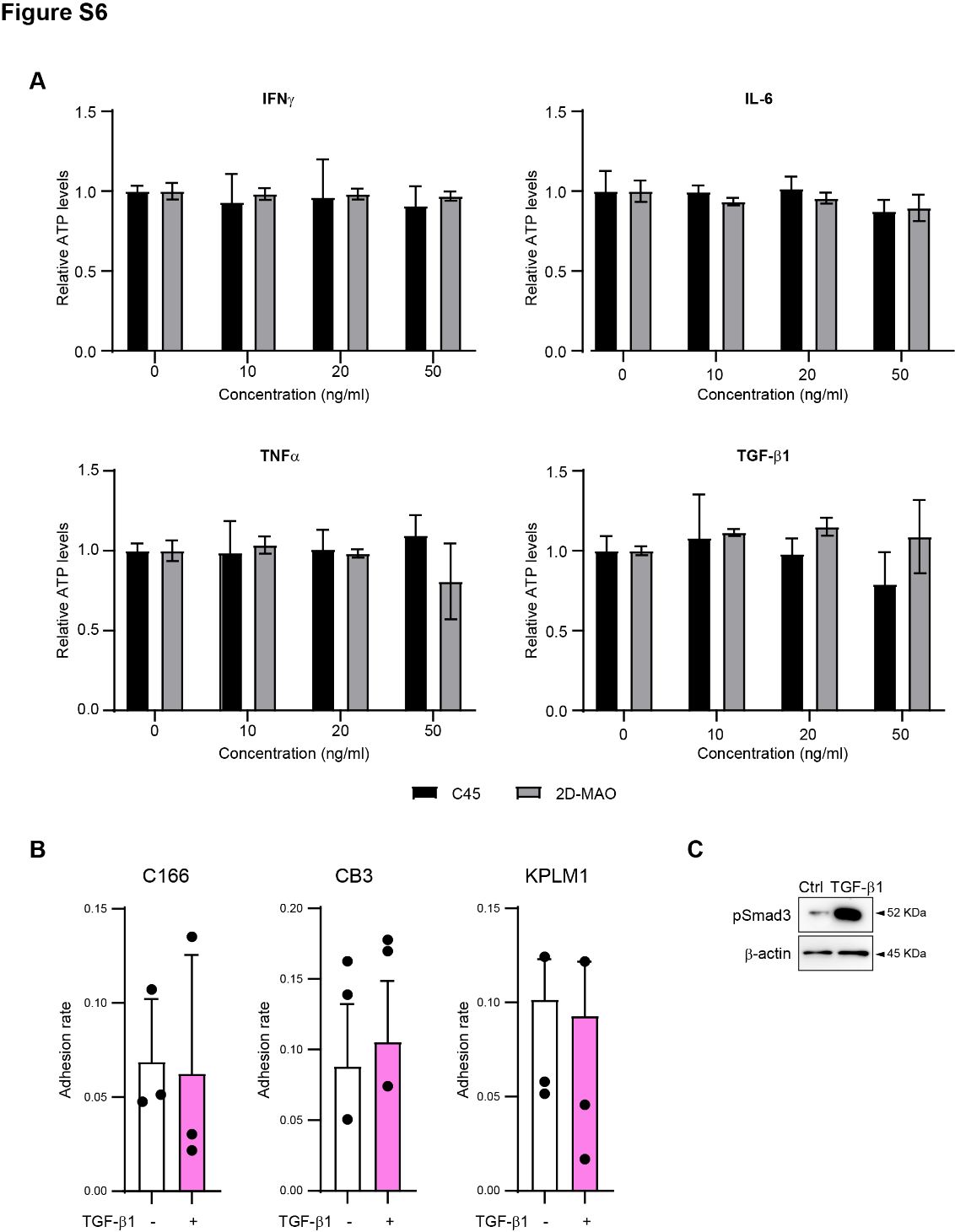


**Figure S6.** Effect of cytokines on the viability and adhesion rates of CRC organoids and 2D-MAOs. (A) Relative ATP values for the indicated doses of cytokines are shown. All of the values for each cytokine are not significant. (B) Adhesion rate of CRC organoids (C166, CB3, KPLM1) to 2D-MAOs after 48 h co-culture. Organoids were pre-treated with TGF-β1 before the assay. (C) Western blotting of phosphorylated Smad3 in 2D-MAOs with or without TGF-β1 treatment.


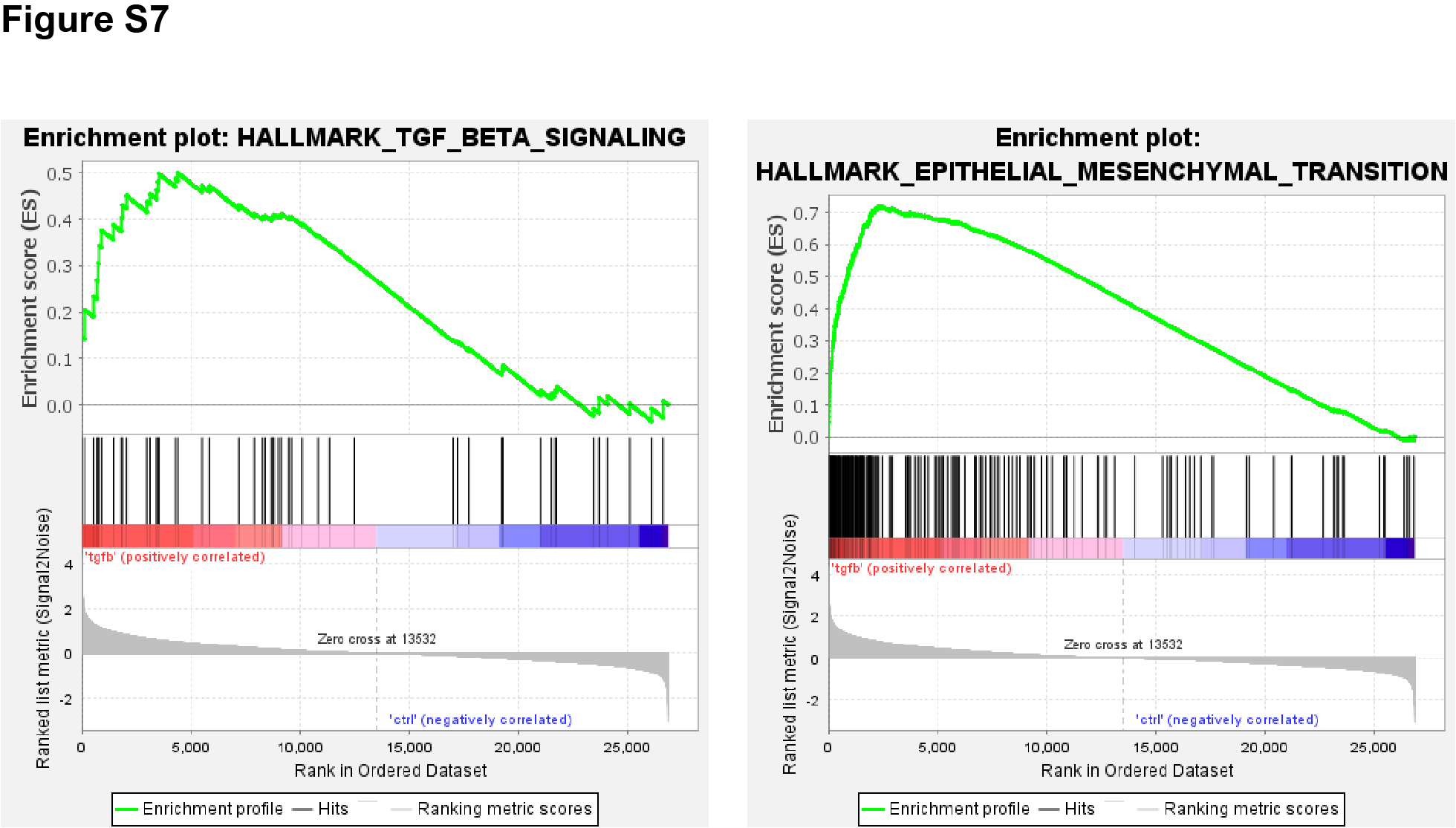


**Figure S7.** Gene set enrichment analysis (GSEA) between TGF-β1-treated and non-treated 2D-MAOs. GSEA of gene signatures for TGF-β1 signalling (left) and epithelial–mesenchymal transition (right).


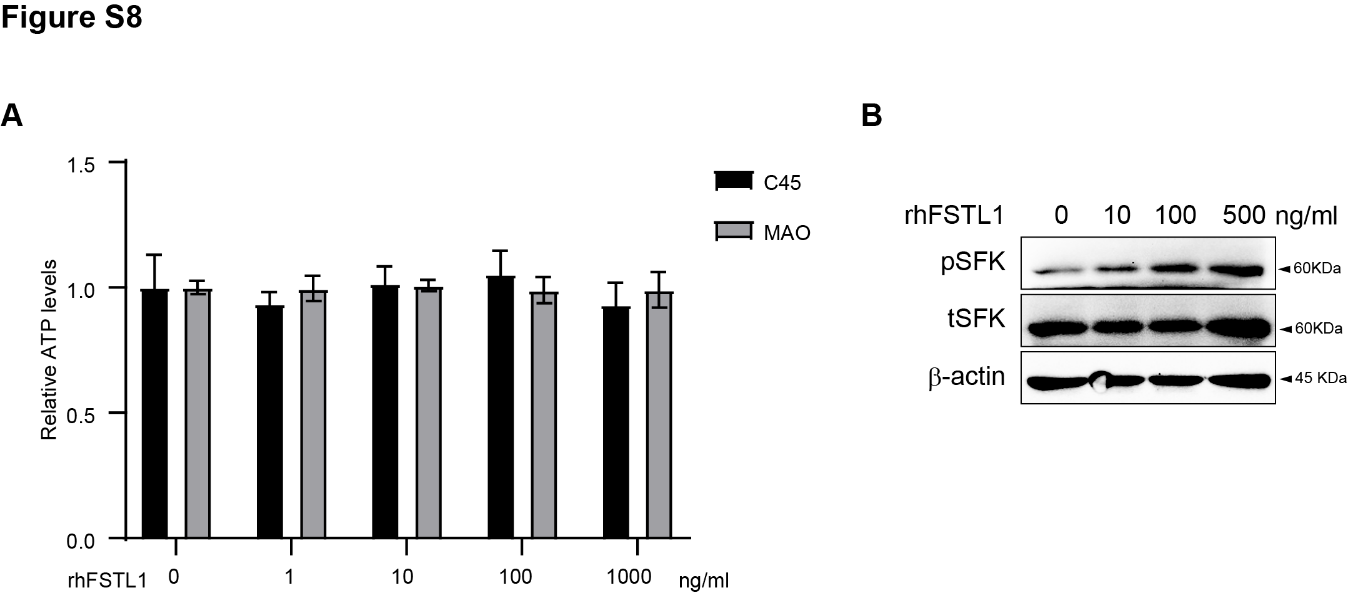


**Figure S8.** Effect of rhFSTL1 on viability and SFK activation. Viability of C45 organoids and 2D-MAOs treated with rhFSTL1. Relative ATP values for the indicated doses of rhFSTL1 are shown. All of the values for each cytokine are not significant. (B) Western blotting of SFK and phosphorylated SFK (pSFK). C45 organoids were treated with the indicated doses of rhFSTL1 for 4 days in serum-free conditions (DMEM/F12 + GlutaMAX).

**Table S1.** List of the reagents used.

| **REAGENT or RESOURCE** | **SOURCE** | **IDENTIFIER** |
| --- | --- | --- |
| **Antibodies** | | |
| Anti-mouse IgG | Cell Signaling Technology, Danvers, MA, USA | Cat #: 7076, RRID: AB_330924 |
| Anti-rabbit IgG | Cell Signaling Technology | Cat #: 7074, RRID: AB_2099233 |
| Villin-1 | Cell Signaling Technology | Cat #: 2369 |
| Ezrin (3C12) | Invitrogen, Carlsbad, CA, USA | Cat #: 35-7300 |
| FSTL1 | Proteintech, Rosemont, IL, USA | Cat #: 20182-1-AP |
| Phospho-Src family (Tyr416) | Cell Signaling Technology | Cat #: 2101 |
| Src (36D10) | Cell Signaling Technology | Cat #: 2109 |
| Phospho-Smad3 (Ser423/425) (C25A9) | Cell Signaling Technology | Cat #:9520 |
| β-Actin (8H10D10) | Cell Signaling Technology | Cat #: 3700 |
| Acetylated α-tubulin | Santa Cruz Biotechnology, Dallas, TX, USA | Cat #: sc-23950 |
| MUC5ac (45M1) | Santa Cruz Biotechnology | Cat #: sc-21701 |
| CC10 (E-11) | Santa Cruz Biotechnology | Cat #: sc-365992 |
| Caveolin-1 (D46G3) | Cell Signaling Technology | Cat #: 3267 |
| Alexa Fluor™ 555 Goat anti-Mouse IgG (H+L) | Thermo Fisher Scientific, Waltham, MA, USA | Cat #: A-21422 |
| Alexa Fluor™ 555 Goat anti-Rabbit IgG (H+L) | Thermo Fisher Scientific | Cat #: A-21428, RRID: AB_2535849 |
| Alexa Fluor™ 488 Goat anti-Rabbit IgG (H+L) | Thermo Fisher Scientific | Cat #: A11008 |
| Alexa Fluor™ 488 Goat anti-Rabbit IgG (H+L) | Thermo Fisher Scientific | Cat #: A-11008, RRID: AB_143165 |
| **Chemicals, peptides, and recombinant proteins** | | |
| Advanced DMEM/F-12 | Thermo Fisher Scientific | 12634-010 |
| Recombinant Human KGF (FGF-7) | Peprotech, Cranbury, NJ, USA | 100-19 |
| Recombinant Human FGF-10 | Peprotech | 100-26 |
| Recombinant Human Noggin | Peprotech | 120-10c |
| Human Interferon Gamma | PBL Assay Science, Piscataway, NJ, USA | 11500-2 |
| Interleukin-6 human | Sigma-Aldrich, St. Louis, MO, USA | I3268 |
| Recombinant Murine TNF-α | Peprotech | 315-01A |
| Transforming Growth Factor-β1 (TGF-β1), Human, recombinant | Fujifilm, Tokyo, Japan | 209-16544 |
| Recombinant Human FSTL1 | Peprotech | 120-51 |
| *N*-Acetyl-l-cysteine | Fujifilm | 015-05132 |
| Nicotinamide | Sigma-Aldrich | N0636 |
| GlutaMAX supplement | Thermo Fisher Scientific | 35050-061 |
| HEPES | Fujifilm | 3242-01375 |
| Dimethyl sulfoxide (DMSO) ReagentPlus®, ≥99.5% | Sigma-Aldrich | D5879 |
| Matrigel® Basement Membrane Matrix | Corning, Corning, NY, USA | 354234 |
| Matrigel® Growth Factor Reduced (GFR) Basement Membrane Matrix | Corning | 354230 |
| Penicillin-Streptomycin (10,000 U/mm) | Thermo Fisher Scientific | 15140122 |
| StemPro™ hESC SFM | Thermo Fisher Scientific | A1000701 |
| Collagenase, Type 4 | Worthington Biochemicals, Lakewood, NJ, USA | LS004188 |
| Cellmatrix Type 1-A | Nitta Gelatin, Osaka, Japan | 651-00653 |
| Endothelial Cell Medium | ScienCell Research Laboratories, Carlsbad, CA, USA | 1001 |
| Bovine plasma fibronectin | ScienCell Research Laboratories | 8284 |
| Trypsin-EDTA (0.25%) | Thermo Fisher Scientific | 25200072 |
| TrypLE Express Enzyme | Thermo Fisher Scientific | 12604-021 |
| Y-27632 | LC Laboratories, Woburn, MA, USA | LCL-Y-5301 |
| A83-01 | Tocris Bioscience, Bristol, UK | 2939 |
| SB202190 | Selleck Chemicals, Houston, TX, USA | S1077 |
| ProLong™ Gold Antifade Mountant with DAPI | Thermo Fisher Scientific | P36931 |
| Hoechst 33342 | Thermo Fisher Scientific | H3570 |
| Rhodamine Phalloidin | Thermo Fisher Scientific | R415 |
| CellTracker™ Red CMTPX Dye | Thermo Fisher Scientific | C34552 |
| CellTracker™ Green CMFDA Dye | Thermo Fisher Scientific | C7025 |
| CellTiter-Glo® Luminescent Cell Viability Assay | Promega, Madison, WI, USA | G755A |
| VivoGlo™ Luciferin | Promega | P1041 |
| **Mice** | | |
| NOD/SCID mice | CLEA Japan, Tokyo, Japan |  |
| C57BL/6J mice | CLEA Japan |  |
| **Experimental Models: Cell Lines** | | |
| Human Hepatic Sinusoidal Endothelial Cells (HHSECs) | ScienCell Research Laboratories | 5000 |
| **Software and Algorithms** | | |
| GraphPad Prism 9 | GraphPad Software Inc., San Diego, CA, USA | [https://www.graphpad.com/](about:blank) |
| GSEA | Broad Institute, Cambridge, MA, USA | [https://www.gsea-msigdb.org/gsea/index.jsp](about:blank) |
| ImageJ (Fiji) | [doi:10.1038/nmeth.2019](about:blank) | [https://imagej.net/software/fiji](about:blank) |
| LAS X Life Science | Leica Microsystems, Wetzlar, Germany | [https://www.leica-microsystems.com/products/microscope-software/p/leica-las-x-ls/downloads/](about:blank) |
| CellSens | Olympus, Tokyo, Japan | https://www.olympus-lifescience.com/en/software/cellsens/ |
| **Other** | | |
| Falcon® Cell Strainers | Corning | 352340; 352350; 352360 |
| 96-Well flat clear bottom black polystyrene TC-treated microplates | Corning | 3904 |
| Glass bottom dish | Matsunami, Osaka, Japan | D11530H |
